# Dicarboxylate recognition based on ultracycle hosts through cooperative hydrogen bonding and anion–π interactions

**DOI:** 10.3762/bjoc.21.72

**Published:** 2025-05-06

**Authors:** Wen-Hui Mi, Teng-Yu Huang, Xu-Dong Wang, Yu-Fei Ao, Qi-Qiang Wang, De-Xian Wang

**Affiliations:** 1 Beijing National Laboratory for Molecular Sciences, CAS Key Laboratory of Molecular Recognition and Function, Institute of Chemistry, Chinese Academy of Sciences, Beijing 100190, China; 2 University of Chinese Academy of Sciences, Beijing 100049, China

**Keywords:** anion–π interactions, anion recognition, hydrogen bonding, dicarboxylates, ultracycles

## Abstract

The efficient binding of dicarboxylates represents an important yet challenging issue in supramolecular chemistry. In this study, we designed functional ultracycles as hosts to accommodate large organic dicarboxylate anions. These ultracycles were synthesized via a one-pot strategy starting from macrocyclic precursors. Host–dicarboxylate binding was investigated using ^1^H NMR titrations, revealing that **B4aH** exhibits strong binding affinities toward a series of dicarboxylates, with association constants reaching up to 6896 M^−1^. The selectivity for heptanedioate (**C7^2−^**) was attributed to cooperative hydrogen bonding, anion–π interactions, and a size-matching effect, as supported by DFT optimizations.

## Introduction

Macrocycles containing more than 50 atoms in the macrocyclic skeleton are denoted as ultracycles [1]. These very large macrocycles are prevalent in nature and exhibit unique functions. For instance, the archaeal lipid GDGT-0 enables archaea to thrive in extreme environments [2]; cycloamyloses enhance the stability of drug metabolism [3–4]; cyclic peptides play critical roles in plant or bacterial defenses and as well as animal hormone signaling [5–6]; cyclic proteins exhibit diverse therapeutic functions [7]; and cyclic nucleotides are essential for molecular cloning and hold potential for disease treatment [8]. In contrast, synthetic ultracycles remain relatively unexplored due to the significant synthetic challenges [9–19]. Among these, very large macrocycles constructed from smaller macrocyclic building units are particularly underexplored. Such macrocycle-containing ultracycles are anticipated to exhibit high association efficiency and selectivity for large guests, driven by cooperative effects of their convergent macrocyclic elements.

Dicarboxylates are crucial species in biological system and chemistry [20]. Examples such as malonate, succinate, and glutarate play key roles in cellular metabolism; they regulate the activity of numerous enzyme receptors, and serve as intermediates in the synthesis of more complex biomolecules [21]. However, excessive consumption or production, as well as insufficient clearance of dicarboxylates, can lead to various health problems [22]. Additionally, dicarboxylates like tartrate, adipate or citrate are widely used as food additives [23–24]. Furthermore, functional materials based on dicarboxylates are expected to play a significant role in future technologies [25]. Therefore, the recognition and detection of dicarboxylates are of great importance. Despite the development of a number of receptors for dicarboxylates [21,26–27], their recognition remains a challenging task due to their strong hydrophilicity (−400 kJ/mol) [28–29], dispersed negative charges at both ends, complex shapes, and flexible conformations. Moreover, the similar carbon skeletons of many dicarboxylates make selective recognition particularly difficult. To address these challenges, we envisioned that ultracycles composed of macrocycles with anion-binding capabilities could serve as suitable hosts for efficient and selective dicarboxylate recognition. In this study, we report the design of ultracycles constructed from functional tetraoxacalix[2]arene[2]triazine submacrocycles. These submacrocycles feature hydroxy groups as hydrogen-bonding (HB) donors on the lower rim, which, in combination with electron-deficient triazines, create cooperative HB and anion–π binding sites to enhance anion binding [30]. The recognition capabilities of these ultracycles toward a range of dicarboxylates were successfully demonstrated.

## Results and Discussion

### Synthesis and structure

**Scheme 1:**
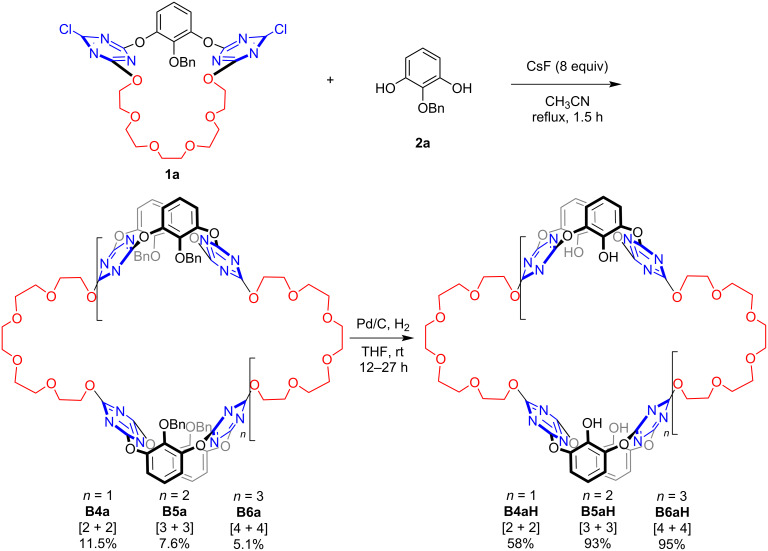
Synthesis of ultracycles.

The ultracycles **B4aH**, **B5aH**, and **B6aH** were synthesized following the previously reported procedure [29,31]. A one-pot reaction of submacrocycle **1a** and 2-(benzyloxy)benzene-1,3-diol (**2a**) in the presence of 8.0 equivalents of CsF yielded the ultracycle precursor compounds. Three reorganized products **B4a**, **B5a**, and **B6a** with different ring sizes were isolated. As previously observed, the structural reorganization likely involves the cleavage and re-formation of the dynamic C_triazine_–OAr bonds, and the presence of an excess of base could facilitate the formation of the thermodynamic-favored reorganized products [29,31]. The benzyl groups were subsequently removed under Pd/C and H_2_ conditions to afford the target ultracyclic hosts. The synthesized ultracycles were fully characterized by spectrometric and elemental analysis (Scheme 1 and Schemes S1‒S3 in Supporting Information File 1).

**Figure 1:**
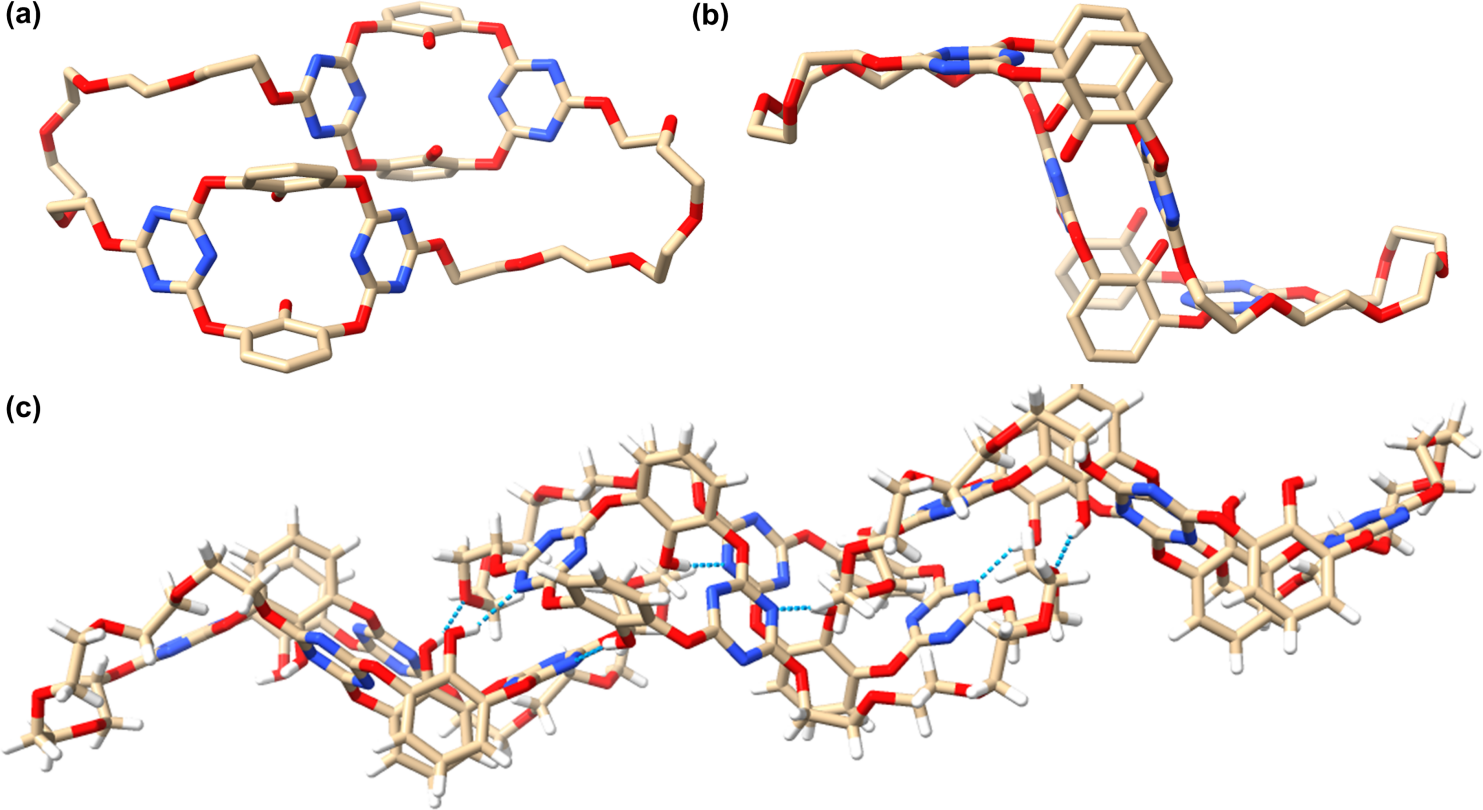
(a, b) Crystal structure of **B4aH** (hydrogen atoms are omitted for clarity), and (c) the stacking structure of the crystal of **B4aH** (the blue dotted lines represent hydrogen bonds).

Single crystals of **B4aH** were obtained by slow vapor diffusion of ethyl ether into an acetonitrile/chloroform 1:1 (v/v) solution of the compound at 4 °C, enabling structural analysis of the ultracycle. As illustrated in Figure 1, the backbone of **B4aH** adopts a Z-like shape with a flexible conformation. The two oxacalix[2]arene[2]triazine subcavities are positioned along the short axis in a staggered face-to-face arrangement, while the glycol chains are oriented along the long axis in the opposite orientations. In packing mode, the submacrocycle units form close contacts through intermolecular hydrogen bonding, C–H···π, and lone pair–π interactions, resulting in a 1D linear assembly.

### Anion recognition

**Figure 2:**
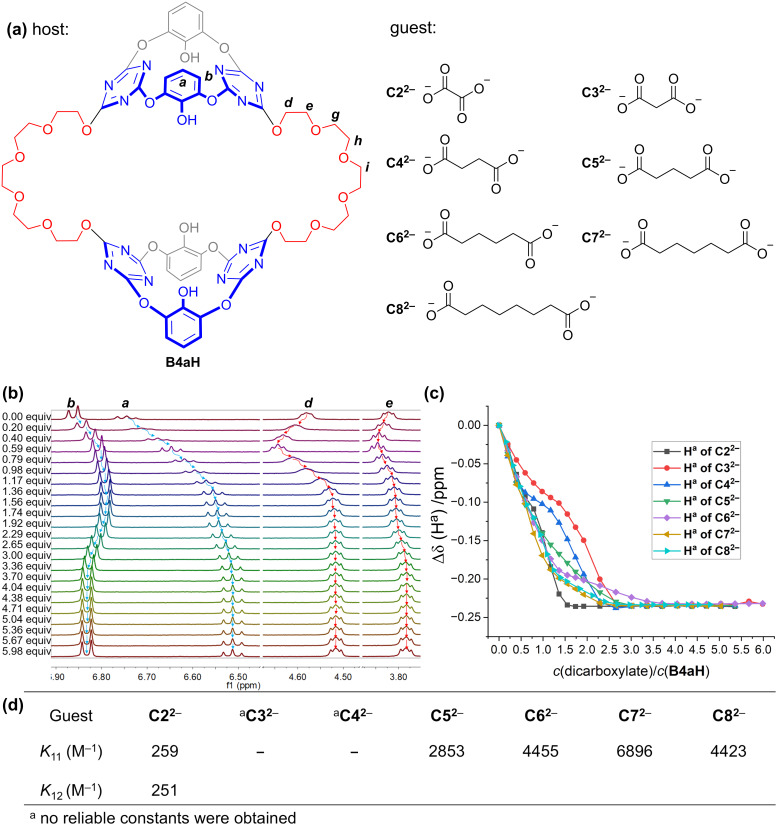
(a) The structures of host and guests, (b) ^1^H NMR spectra (298 K, 400 MHz, CD_3_CN) of **B4aH** upon titration with **C6^2−^** (*c*(**B4aH**) = 1 mM), (c) chemical shift changes of H^a^ versus titration equivalents *c*(dicarboxylate)/*c*(**B4aH**), and (d) association constants of host and guests.

With the functional ultracycles in hand, we investigated the binding between the [2 + 2] ultracycle **B4aH**, which contains two electron-deficient cavities, and a series of dicarboxylate anions (**C2^2−^**–**C8^2−^** as tetrabutylammonium salts) by ^1^H NMR titration experiments (Figure 2). Taking **C6^2−^** as an example, when it was added dropwise to a solution of **B4aH**, the aromatic proton H^a^ exhibited continuous upfield shifts, while H^b^ initially shifted upfield and then downfield upon the addition of 1.5 equiv of **C6^2−^** (Figure 2b and Figure S8 in Supporting Information File 1). These chemical shift changes indicate the interaction between the carboxylate heads and the submacrocycles. Additionally, the protons H^d^ and H^e^ on the glycol chain showed initial downfield shifts followed by upfield shifts. These discontinuous chemical shift movements suggest the host–dicarboxylate interactions and the simultaneous conformational changes in the host upon guest inclusion. Similar chemical shift changes of **B4aH** were observed for other dicarboxylates, indicating a consistent binding mode across the series (Figure 2b and c and Figures S4–S10 in Supporting Information File 1).

The titration curves (H^a^) were analyzed using the Bindfit program [32–34] to determine the binding constants of **B4aH** with guests. A 1:1 binding stoichiometry best fit the titration curves for **C5^2−^**–**C8^2−^**, with binding constants following the order of **C7^2−^** > **C8^2−^** ≈ **C6^2−^** > **C5^2−^**, suggesting a dependence on the length of the dicarboxylates (Figure 2c and d). We proposed that the dicarboxylates interact with each subcavity of **B4aH** through their terminal anionic groups, utilizing cooperative hydrogen bonding and anion–π interactions. The optimal size matching between dicarboxylate and the host cavity, as seen with **C7^2−^**, enhances the synergistic effect between the two subcavities, resulting in a higher binding strength. Dicarboxylates longer or shorter than **C7^2−^** (e.g., **C8^2−^**, **C6^2−^**, or **C5^2−^**) exhibit weaker binding due to their less-matched host–guest sizes. For the shorter dicarboxylates (**C2^2−^**–**C4^2−^**), the binding behavior is more complex. For instance, the [**B4aH·C2^2−^**] complex fits a 1:2 binding model, with two-step binding constants of *K*_11_ = 259 M^−1^ and *K*_12_ = 251 M^−1^, implying that **B4aH** can accommodate two **C2^2−^** anions as a dimer within its cavity [29]. Malonate (**C3^2−^**) and succinate (**C4^2−^**) exhibited irregular titration curves (Figure 2c), and no reliable binding constants could be obtained using either 1:1 or 1:2 binding models. This is likely due to their intermediate size of chain lengths, which are neither long enough for 1:1 binding nor capable of squeezing a dimer for 1:2 complexation. Notably, the unsubstituted ultracycle **B4** [31] without the pendant OH groups on the lower rim, which relies on solely anion–π interactions, showed weak binding affinity for **C6^2−^** (Figure S11 in Supporting Information File 1). This underscores the importance of cooperative hydrogen bonding and anion–π interactions for the efficient dicarboxylate binding.

**Figure 3:**
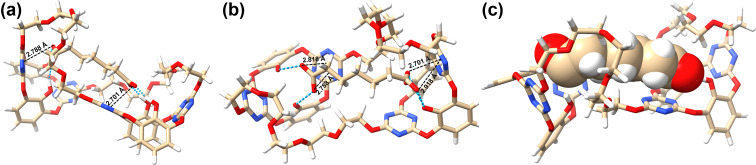
(a–c) DFT-optimized structure of the **B4aH-C7^2−^** complex. The blue dotted lines represent hydrogen bonds and the black dotted lines represent anion–π interactions.

To visualize the proposed synergistic hydrogen bonding and anion–π interactions between the host and guest, we carried out geometry optimizations using M06-2X at the 6-31G(d) level of theory, taking the [**B4aH·C7^2−^**] complex as a representative example [35–36]. The optimized structure, shown in Figure 3, reveals a 1:1 complex in which the dicarboxylate **C7^2−^** is included within the cavity of **B4aH**. The two subcavities interact synergistically with the included dicarboxylate; the two terminal carboxylate groups are respectively positioned within electron-deficient cavities of the submacrocycles, forming hydrogen bonds (2.49–2.59 Å) with the two hydroxy groups and engaging in anion–π interactions (2.70–2.92 Å) with the triazine rings (Figure 3). Additionally, the glycol arms of the macrocycle may further stabilize the anion binding through van der Waals interactions with the alkyl chains of the dianions. Driven by these multiple noncovalent interactions, the host undergoes conformational adjustments: the distance between the two submacrocycles increases, and the glycol chains adopt extended conformations compared to the structure shown in Figure 1. These results suggest that the experimentally observed strong binding capability and selectivity of **B4aH** for **C7^2−^** likely arise from cooperative noncovalent interactions and a size-matching effect.

## Conclusion

In conclusion, we synthesized a series of hydroxy-substituted ultracycles of varying sizes on the lower-rim using a one-pot cyclization strategy. ^1^H NMR titration experiments indicate that the introduction of lower-rim hydroxy substituents effectively enhances the dicarboxylate binding through cooperative hydrogen bonding and anion–π interactions. The selective recognition of long and flexible dicarboxylates holds exciting promise for the use of dicarboxylate sensors in medicine and industry.

## Supporting Information

File 1 Experimental details and characterization data (including ^1^H NMR, ^13^C NMR, IR, and HRMS of precursor compounds and ultracycles, X-ray data for **B4aH**, theoretical calculations, and NMR titration data).

## Data Availability

All data that supports the findings of this study is available in the published article and/or the supporting information of this article.
