# Supplementary material for: Dicarboxylate recognition based on ultracycle hosts through cooperative hydrogen bonding and anion–π interactions
Source: Beilstein J Org Chem. 2025 May 6;21:884–9. doi: 10.3762/bjoc.21.72 (PMC12067094; doi:10.3762/bjoc.21.72)
Supplement: File 1 — Experimental details and characterization data (including 1H NMR, 13C NMR, IR, and HRMS of precursor compounds and ultracycles, X-ray data for B4aH, theoretical calculations, and NMR titration data). [file Beilstein_J_Org_Chem-21-884-s001.pdf]

BEILSTEIN JOURNAL OF ORGANIC CHEMISTRY

Supporting Information

for

Dicarboxylate recognition based on ultracycle hosts through cooperative hydrogen bonding and anion–π interactions

Wen-Hui Mi, Teng-Yu Huang, Xu-Dong Wang, Yu-Fei Ao, Qi-Qiang Wang and De-Xian Wang

Beilstein J. Org. Chem. **2025,** *21,* 884–889. doi:10.3762/bjoc.21.72

Experimental details and characterization data (including ^{1}H NMR, ^{13}C NMR, IR, and HRMS of precursor compounds and ultracycles, X-ray data for B4aH, theoretical calculations, and NMR titration data)

License and Terms: This is a supporting information file under the terms of the Creative Commons Attribution License (https://creativecommons.org/licenses/by/4.0). Please note that the reuse, redistribution and reproduction in particular requires that the author(s) and source are credited and that individual graphics may be subject to special legal provisions.
The license is subject to the *Beilstein Journal of Organic Chemistry* terms and conditions: (https://www.beilstein-journals.org/bjoc/terms)

Table of contents

1. General information S2

2. Synthesis and characterization S3

2.1 Synthesis and characterization of macrocyclic precursors S3

2.2 Synthesis and characterization of ultracyclic precursors S4

2.3 HRESIMS studies of ultracyclic precursors. S6

2.4 Debenzylation of ultracycles. S7

3. ^{1}H NMR titrations S9

3.1 Titration method and analysis S9

3.2 Titration data S9

3.2.1 Titration between **B4aH** and dicarboxylates S9

3.2.2 Titration between **B4** and dicarboxylates. S17

4. X-Ray diffraction data S18

5. DFT calculation S20

6. Copies and ^{1}H and ^{13}C NMR spectra S23

7. Reference S30

1. General information

All chemicals were obtained from commercial sources and used without further purification unless stated otherwise. NMR spectra were recorded on Bruker 400 and 500 MHz NMR spectrometers at room temperature. Chemical shifts are reported in ppm and referenced to tetramethylsilane or the residual solvent resonance. Mass spectra were measured on a Thermo Fisher Exactive Mass Spectrometer. Infrared spectra were recorded using a Nicolet-6700 FT-IR spectrometer with KBr pellets in the 4000–400 cm^{-1} region. Elemental analysis was recorded on Carlo Erba 1106. Melting points were determined on Melting Point Instrument SGWX–4 using a microscopic hot plate method and are uncorrected. X-ray diffraction was performed on a Rigaku R-AXIS RAPID IP.

2. Synthesis and characterization

2.1 Synthesis and characterization of macrocyclic precursors

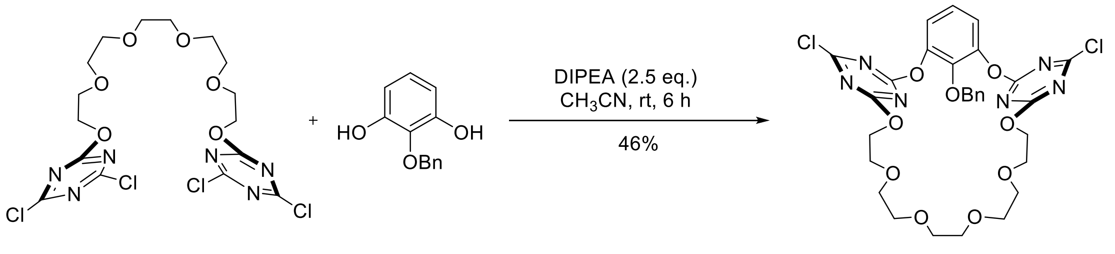

Scheme S1. Synthesis of macrocyclic precursor **1a**.

Synthesis of **1a** (in a manner similar to our previously reported procedure[S1]). Compound **3a**[S2] (1.07g, 2.00 mmol) and 2-(benzyloxy)benzene-1,3-diol (**2a**[S3], 0.44 g, 2.00 mmol) were dissolved in 200 mL acetonitrile. To this solution was added dropwise DIPEA (0.65 g, 5.00 mmol) in 60 mL acetonitrile during a period of 0.5 h, and then kept stirring for 5.5 h, and then solvent was removed under vacuum by a rotary evaporator. The residue was chromatographed on silica gel (100–200 mesh) with a mixture of ethyl acetate and petroleum ether 1:1 (v/v) as eluent to yield **1a** (0.63 g, 46%) as white solid.

1a: mp 38–41 °C; ^{1}H NMR (DMSO-d_6, 500 MHz) δ (ppm) 7.37–7.21 (m, 6H), 7.12 (s, 2H), 4.92 (s, 2H), 4.38 (t, J = 4.4 Hz, 4H), 3.67 (t, J = 4.6 Hz, 4H), 3.46 (m, 12H); ^{13}C NMR (DMSO-d_6, 125 MHz) δ (ppm) 172.4, 172.2, 171.8, 145.2, 142.4, 136.6, 128.6, 128.5, 127.8, 124.5, 121.6, 75.5, 70.3, 68.9, 68.2; IR (KBr) ν 3201, 3059, 2877, 2803, 1700, 1455, 1397, 1094, 1060, 545 cm^{-1}; HR-ESI-MS (positive ion mode) m/z: $[M + H]^+$ calcd for $C_{29}H_{31}Cl_2N_6O_9$: 677.1524, found: 677.1518; Anal. calcd (%) for $C_{29}H_{30}Cl_2N_6O_9$ + H_2O: C, 48.82; H, 4.80; N, 11.78, found: C, 48.89; H, 4.96; N, 11.73.

2.2 Synthesis and characterization of ultracyclic precursors

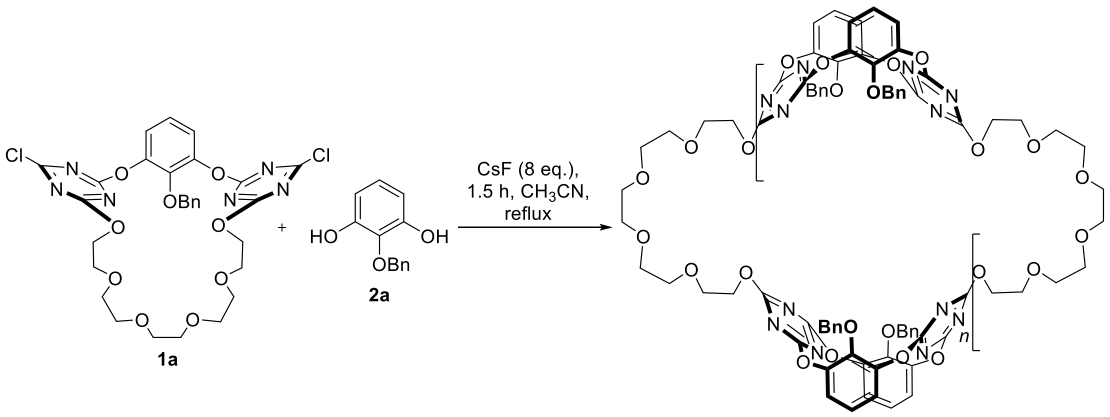

n = 1	*n* = 2	*n* = 3
**B4a**	**B5a**	**B6a**
[2+2]	[3+3]	[4+4]
6.3-11.5%	4.2-7.6%	3.8-5.1%

Scheme S2. Synthesis of ultracyclic precursors **B4a**, **B5a** and **B6a**.

Synthesis of ultracycles **B4a**, **B5a**, and **B6a**. Compound **1a** (1.88 g, 3.00 mmol), 2-(benzyloxy)benzene-1,3-diol (**2a**, 0.65 g, 3.00 mmol) and cesium fluoride (3.65 g, 24.00 mmol) were added into 300 mL dry acetonitrile. Then, the mixture was allowed to reflux for 1.5 h under argon atmosphere. After cooling to room temperature, the mixture was filtered, and the filtrate was concentrated under vacuum by a rotary evaporator and then chromatographed on silica gel (100–200 mesh) with a mixture of dichloromethane/acetone 6:1–4:1 as eluent to yield **B4a** (155 mg, 6.3%), **B5a** (125 mg, 5.1%), and **B6a** (105 mg, 4.3%) as white solids.

B4a: mp 51−53 °C; ^{1}H NMR (d_6-DMSO, 500 MHz) δ (ppm) 7.07–6.99 (m, 24H), 6.91 (d, J = 7.2 Hz, 8H), 4.72 (s, 8H), 4.39 (t, J = 4.5 Hz, 8H), 3.70 (t, J = 4.5 Hz, 8H), 3.57–3.53 (m, 24H); ^{13}C NMR (DMSO-d_6, 125 MHz) δ (ppm) 174.0, 173.1, 145.3, 142.7, 136.6, 128.3, 128.0, 127.2, 124.2, 121.1, 74.9, 70.4, 70.3, 70.3, 68.4, 68.2; IR (KBr) v 2876, 1577, 1557, 1486, 1382, 1335, 1281, 1238, 1119, 814, 750 cm^{-1}; HR-ESI-MS (positive ion mode) m/z: [M + Na]$^+$ calcd for $C_{84}H_{80}N_{12}O_{24}Na$: 1663.5301, found: 1663.5314; Anal. Calcd (%) for $C_{84}H_{80}N_{12}O_{24}$: C, 61.46; H, 4.91; N, 10.24, found: C, 61.19; H, 4.90; N, 10.09.

B5a: mp 47−52 °C; ^{1}H NMR (CD_3CN, 500 MHz) δ (ppm) 7.12–7.06 (m, 18H), 6.97–6.93 (m, 18H), 6.89 (d, J = 7.8 Hz, 12H), 4.76 (s, 12H), 4.42 (t, J = 4.7 Hz, 12H), 3.74 (t, J = 4.7 Hz, 12H), 3.67–3.54 (m, 39H); ^{13}C NMR (CD_3CN, 125 MHz) δ (ppm) 173.6, 145.9, 143.2, 137.1, 128.6, 128.4, 127.9, 124.0, 75.3, 70.8, 70.8, 70.7, 68.9, 68.5; IR (KBr) v 2922, 1577, 1557, 1486, 1382, 1334, 1281, 1119, 815 cm^{-1}; HR-ESI-MS (positive ion mode) m/z: $[M + 2Na]^{2+}$ calcd for $C_{126}H_{120}N_{18}O_{36}Na_2$: 1253.8965, found: 1253.8983; Anal. Calcd (%) for $C_{126}H_{120}N_{18}O_{36}$: C, 61.46; H, 4.91; N, 10.24, found: C,61.63; H, 5.22; N, 9.57

B6a: mp 51–53 °C; ^{1}H NMR (d_6-DMSO, 500 MHz) δ (ppm) 7.02–6.99 (m, 48H), 6.91 (d, J = 7.3 Hz, 14H), 4.74 (s, 16H), 4.41 (t, J = 4.7 Hz, 16H), 3.71 (t, J = 4.3 Hz, 16H), 3.56–3.52 (m, 48H); ^{13}C NMR (DMSO-d_6, 125 MHz) δ (ppm) 172.9, 171.9, 144.2, 141.6, 135.6, 127.3, 127.0, 126.2, 123.0, 120.0, 73.8, 69.25, 69.20, 67.4, 67.2 ; IR (KBr) v 2878, 1577, 1557, 1486, 1383, 1335, 1281, 1238, 1119, 814, 750 cm^{-1}; HR-ESI-MS (positive ion mode) m/z: $[M + 3Na]^{3+}$ calcd for $C_{168}H_{160}N_{24}O_{48}Na_3$: 1117.3520, found: 1117.3538; Anal. Calcd (%) for $C_{168}H_{160}N_{24}O_{48}$: C, 61.46; H, 4.91; N, 10.24, found: C, 61.77; H, 5.08; N, 9.71.

2.3 HRESIMS studies of ultracyclic precursors.

The high-resolution ESI–MS studies were carried out.

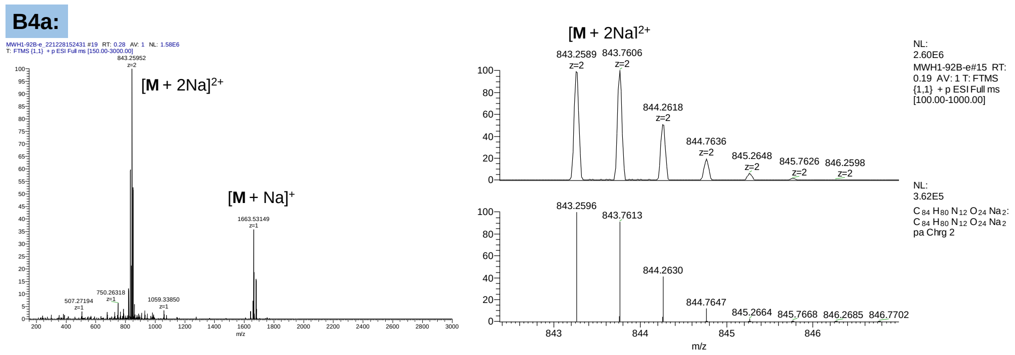

Figure S1. HRESIMS spectra of **B4a**.

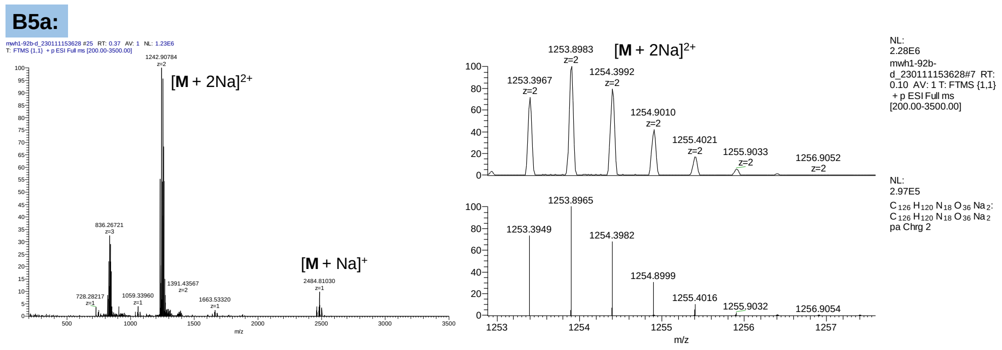

Figure S2. HRESIMS spectra of **B5a**.

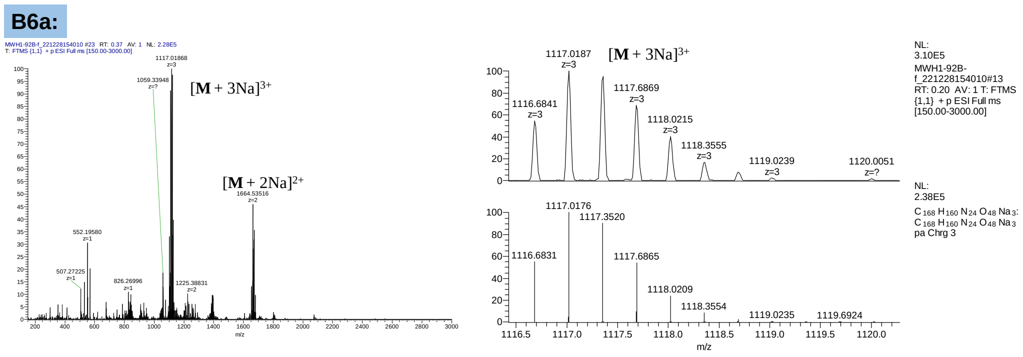

Figure S3. HRESIMS spectra of **B6a**.

2.4 Debenzylation of ultracycles.

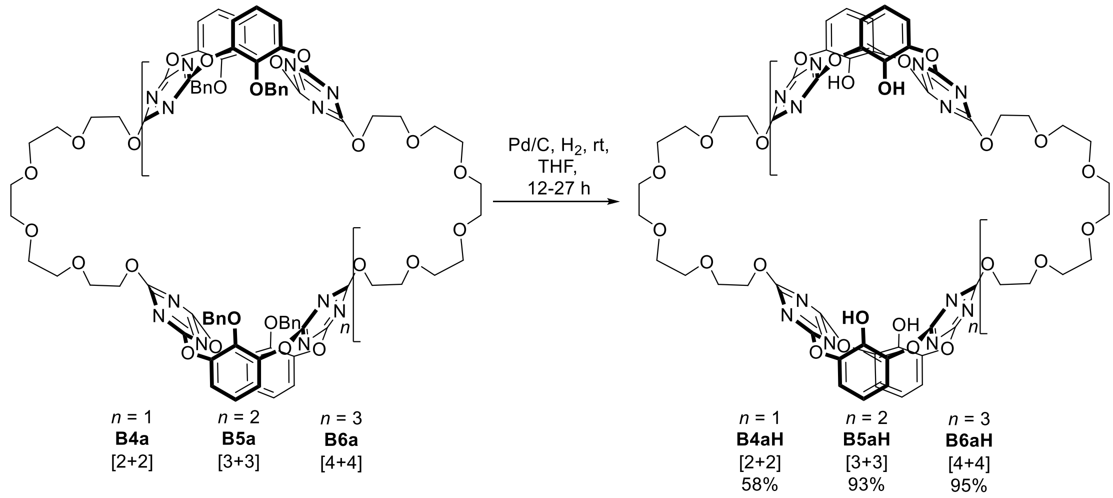

Scheme S3. Debenzylation of ultracycles.

Synthesis of **B4aH** (taking **B4a** as an example, similarly for other compounds). At room temperature, **B4a** (100 mg, 0.0609 mmol), Pd/C (40 mg) and THF (20 mL) were mixed in a two-necked flask. The mixture was stirred for 24 h under a H_2 ballon. After filtration over diatomaceous earth, the filtrate was concentrated under vacuum by a rotary evaporator. The residue was recrystallized from methanol, dichloromethane, acetonitrile and ethyl ether to give purified compound **B4aH** (45 mg, 58%) as white solid.

Table S1. Construction of ultracycles.

Entry	Compound	Amount	Pd/C	Time	Yield
1	**B4a**	0.0609mmol, 100 mg	40 mg	24 h	58%
2	**B5a**	0.0812 mmol, 200 mg	120 mg	27 h	93%
3	**B6a**	0.0609 mmol, 200 mg	150 mg	12 h	95%

B4aH: mp 42–44 ℃; ^{1}H NMR (DMSO-d_6, 500 MHz) δ (ppm) 9.38 (s, 4H), 6.85 (d, J = 8.0 Hz, 8H), 6.63 (t, J = 8.4 Hz, 4H), 4.59 (t, J = 4.7 Hz, 8H), 3.83–3.76 (t, J = 4.0 Hz, 8H), 3.62–3.60 (m, 8H), 3.57–3.55 (m, 16H); ^{13}C NMR (DMSO-d_6, 125 MHz) δ (ppm) 173.9, 173.2, 142.1, 141.2, 120.4, 118.6, 70.3, 70.3, 70.3, 68.65, 67.9; IR (KBr)

ν 2876, 1577, 1500, 1394, 1335, 1259, 1119, 815 cm^{-1}; HR-ESI-MS (negative ion mode) *m*/*z*: $[M - H]^-$ calcd for $C_{56}H_{55}N_{12}O_{24}$: 1279.3447, found: 1279.3468; Anal. calcd (%) for $C_{56}H_{56}N_{12}O_{24}$: C, 52.50; H, 4.41; N, 13.12, found: C, 52.05; H, 4.44; N, 12.73.

B5aH: mp 47–49 °C; ^{1}H NMR (DMSO-d_6, 500 MHz) δ (ppm) 9.44 (s, 6H), 6.83 (d, *J* = 8.1 Hz, 12H), 6.62 (t, *J* = 7.8 Hz, 8H), 4.55 (s, 12H), 3.79 (s, 12H), 3.64–3.52 (m, 36H); ^{13}C NMR (DMSO-d_6, 125 MHz) δ (ppm) 173.3, 172.5, 141.5, 140.4, 119.8, 117.9, 69.7, 69.7, 69.6, 68.1, 67.4; IR (KBr) *ν* 2875, 1578, 1499, 1392, 1336, 1259, 1215, 1120, 845, 816 cm^{-1}; HR-ESI-MS (negative ion mode) *m*/*z*: $[M – H]^-$ calcd for $C_{84}H_{83}N_{18}O_{36}$: 1919.5212, found: 1919.5216; Anal. Calcd (%) for $C_{84}H_{84}N_{18}O_{36} + H_2O$: C, 50.38; H, 4.68; N, 12.59, found: C, 50.82; H, 4.46; N, 12.30.

B6aH: mp 46–48 °C; ^{1}H NMR (DMSO-d_6, 500 MHz) δ (ppm) 9.45 (s, 8H), 6.83 (d, *J* = 7.4 Hz, 16H), 6.63 (t, *J* = 7.7 Hz, 8H), 4.55 (s, 16H), 3.79 (s, 16H), 3.66–3.50 (m, 48H); ^{13}C NMR (DMSO-d_6, 125 MHz) δ (ppm) 173.9, 173.1, 142.1, 141.0, 120.4, 118.5, 70.3, 70.3, 68.6, 67.9; IR (KBr) *ν* 2875, 1578, 1500, 1394, 1336, 1120, 947, 845, 816 cm^{-1}; HR-ESI-MS (positive ion mode) *m*/*z*: $[M + 2Na]^{2+}$ calcd for $C_{112}H_{112}N_{24}O_{48}Na_2$: 1304.3456, found: 1304.3411; Anal. calcd (%) for $C_{112}H_{112}N_{24}O_{48} + H_2O$: C, 50.89; H, 4.61; N, 12.72; found: C, 51.27; H, 4.78; N, 12.03.

3. ^{1}H NMR titrations

3.1 Titration method and analysis

A stock solution of the host (1 mM in CD_3CN) was prepared. The guest solutions were prepared by dissolving the dicarboxylates into the host stock solution, which resulted in a final concentration of 50 mM. 500 μL of the host stock solution was added to an NMR tube to record an original spectrum, then titration spectra were obtained after the addition of aliquots of guest solutions.

The aromatic proton H^a exhibited continuous upfield shifts, while H^b initially shifted upfield and then downfield. These chemical shift changes indicated the hydrogen bonding and anion–π interactions between the carboxylate heads and the submacrocycles. The titration curves (H^a) were analyzed using the Bindfit v0.5 program[S4–S6] to determine the binding stoichiometry (1:1 or 1:2) and binding constants.

3.2 Titration data

3.2.1 Titration between **B4aH** and dicarboxylates

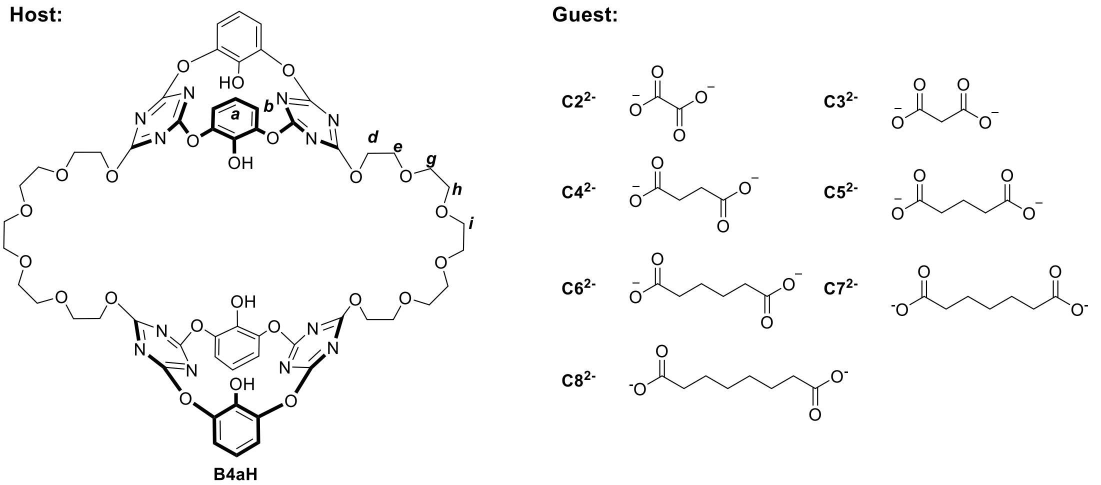

Scheme S4. The structure of **B4aH** and guests.

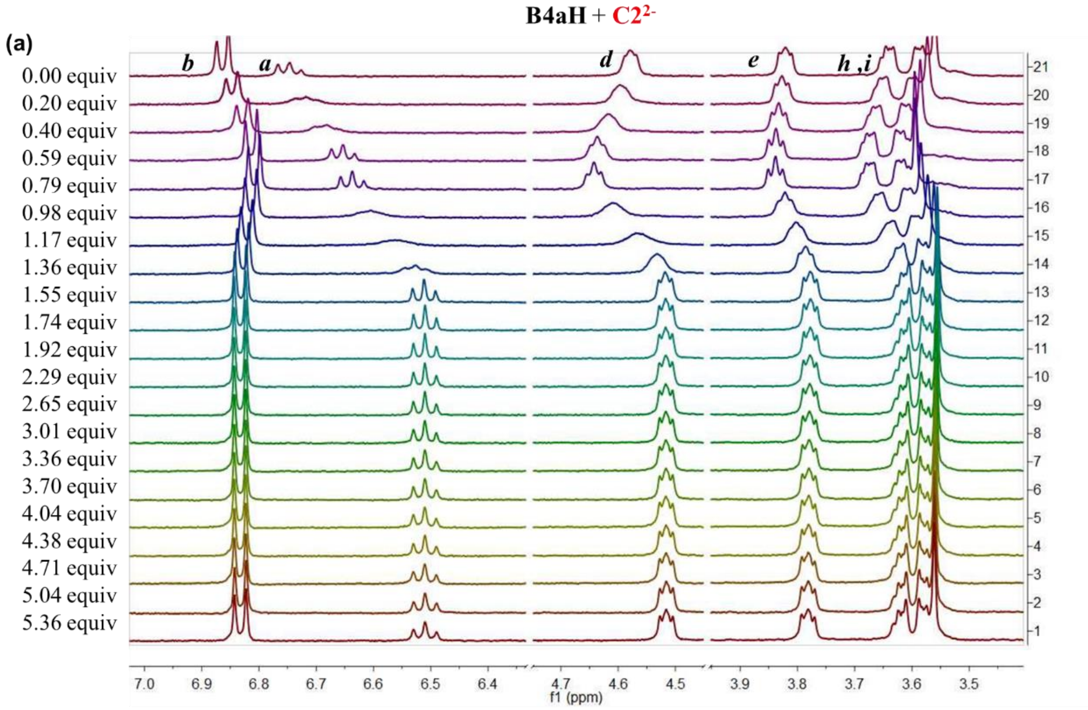

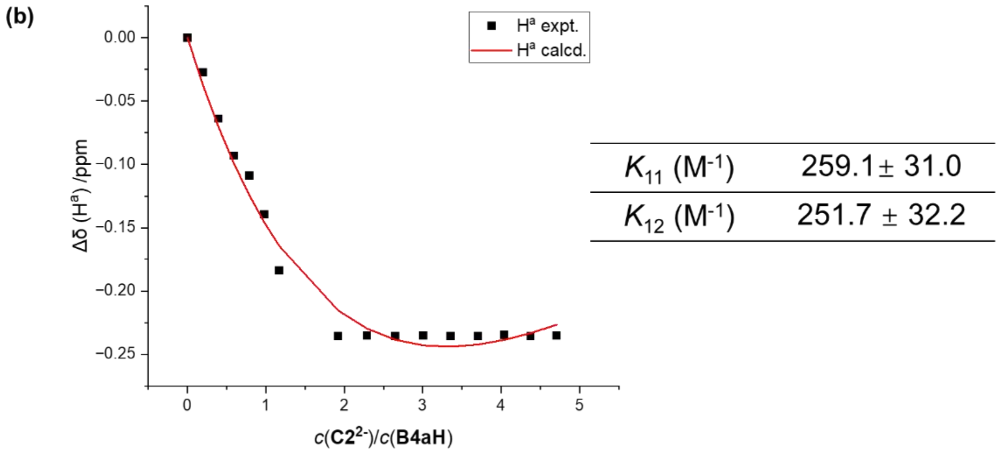

K_{11} (M^{-1})	259.1 ± 31.0
K_{12} (M^{-1})	251.7 ± 32.2

Figure S4. (a) ^{1}H NMR titration between **B4aH** and **C2**$^{2-}$ (c(**B4aH**) = 1 mM, c((**TBA**$^+$)$_2$**C2**$^{2-}$) = 50 mM, 298 K, 400 MHz, CD_3CN); (b) Fitting results of the titration.

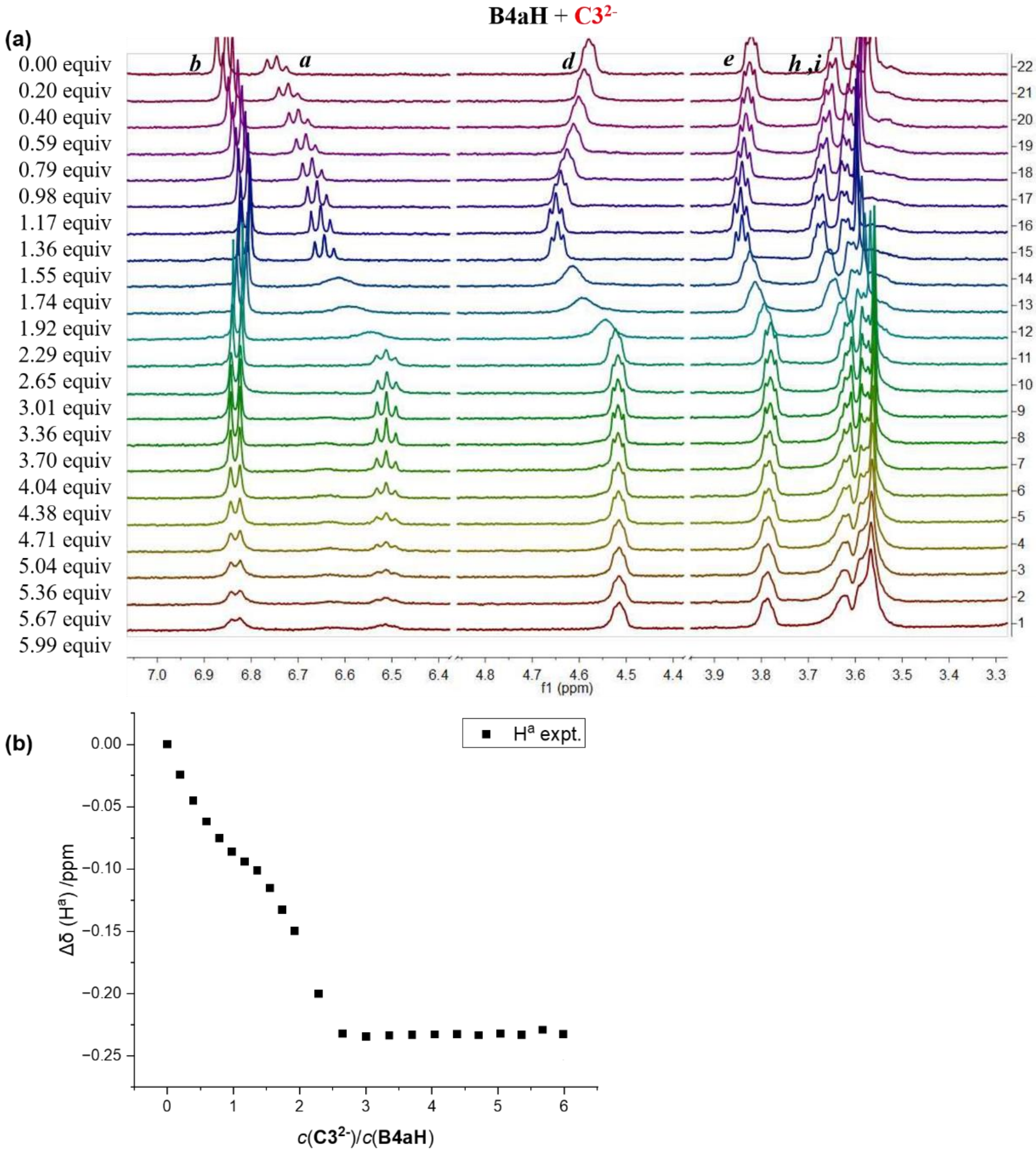

Figure S5. (a) ^{1}H NMR titration between **B4aH** and **C3**$^{2-}$ (c(**B4aH**) = 1 mM, c((**TBA**$^{+}$)$_2$**C3**$^{2-}$) = 50 mM, 298 K, 400 MHz, CD_3CN); (b) the chemical shift change curve. The irregular curve may arise from that the short chain length of the anion prevent synergistic recognition of the two subcavities, and it was also hard for the host to accommodate two anions to reach stable 1:2 complexation. In addition, the host conformation may change upon binding. As a result, no reliable binding constants could be obtained using either 1:1 or 1:2 binding models.

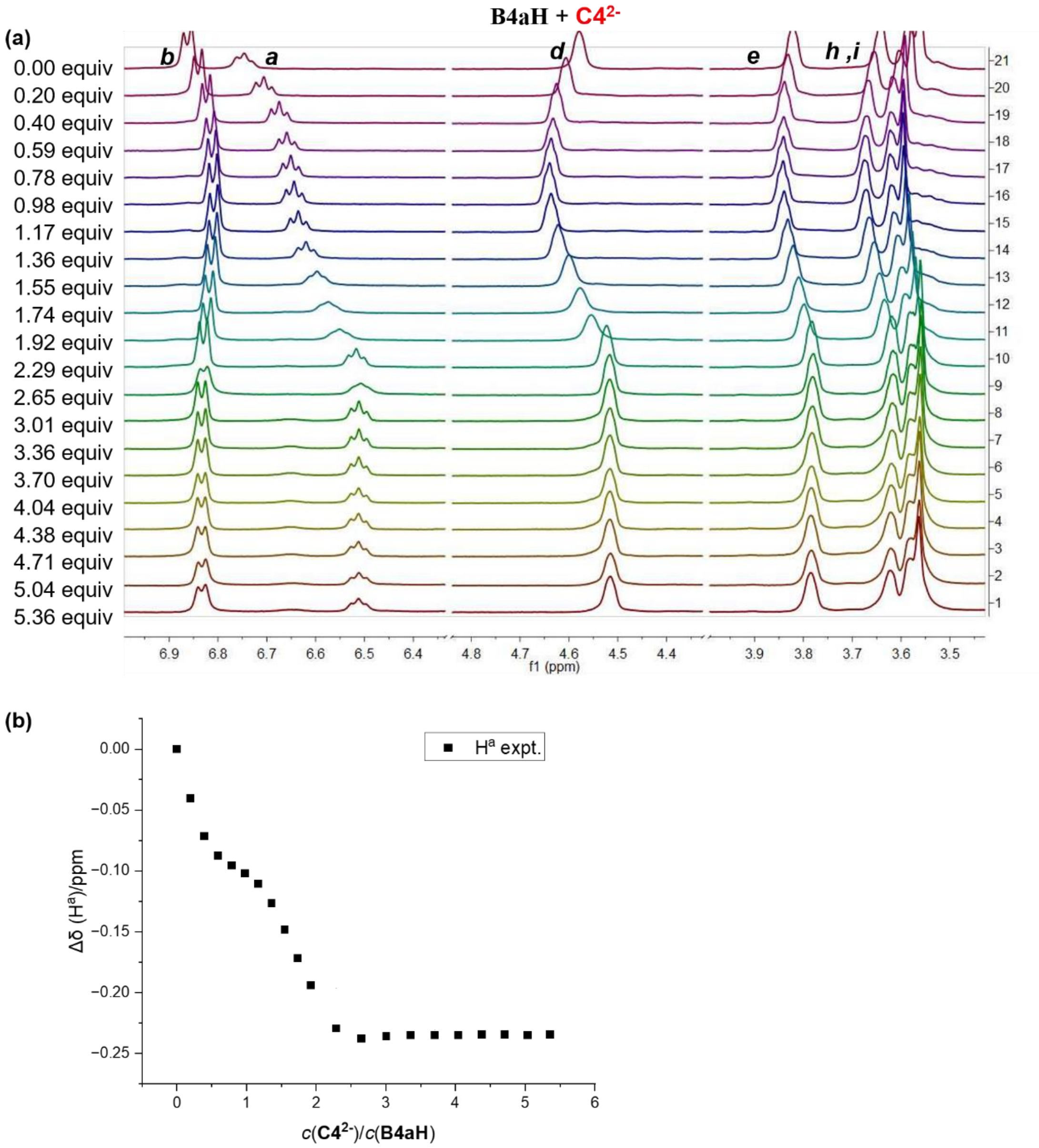

Figure S6. (a) ^{1}H NMR titration between **B4aH** and **C4**$^{2-}$ (c(**B4aH**) = 1 mM, c((**TBA**$^{+}$)$_2$**C4**$^{2-}$) = 50 mM, 298 K, 400 MHz, CD_3CN); (b) the chemical shift change curve. The irregular curve may arise from that the short chain length of the anion prevent synergistic recognition of the two subcavities, and it was also hard for the host to accommodate two anions to reach stable 1:2 complexation. In addition, the host conformation may change upon binding. As a result, no reliable binding constants could be obtained using either 1:1 or 1:2 binding models.

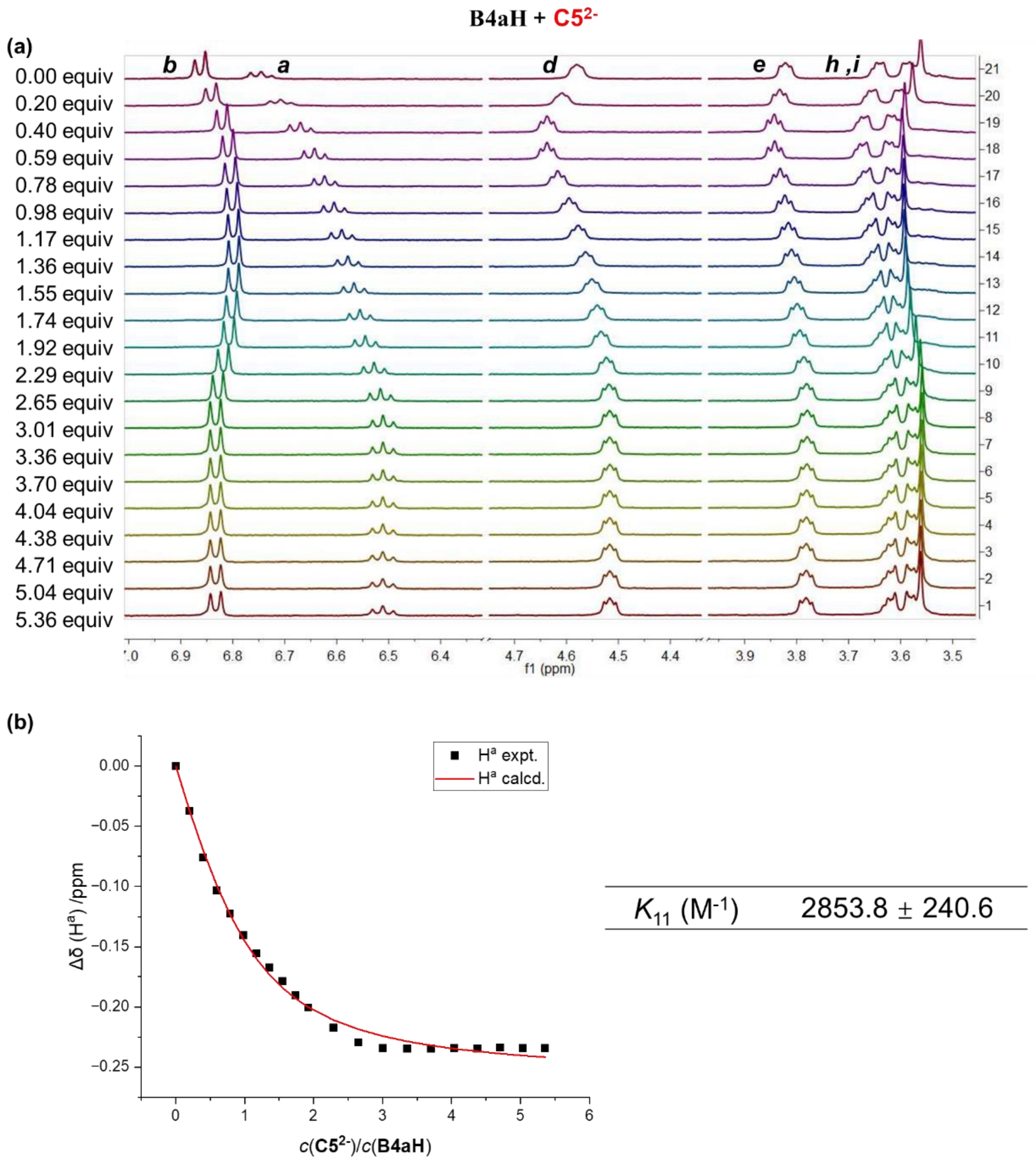

Figure S7. (a) ^{1}H NMR titration between **B4aH** and **C5**$^{2-}$ (c(**B4aH**) = 1 mM, c((**TBA**$^{+}$)$_2$**C5**$^{2-}$) = 50 mM, 298 K, 400 MHz, CD_3CN); (b) Fitting results of the titration.

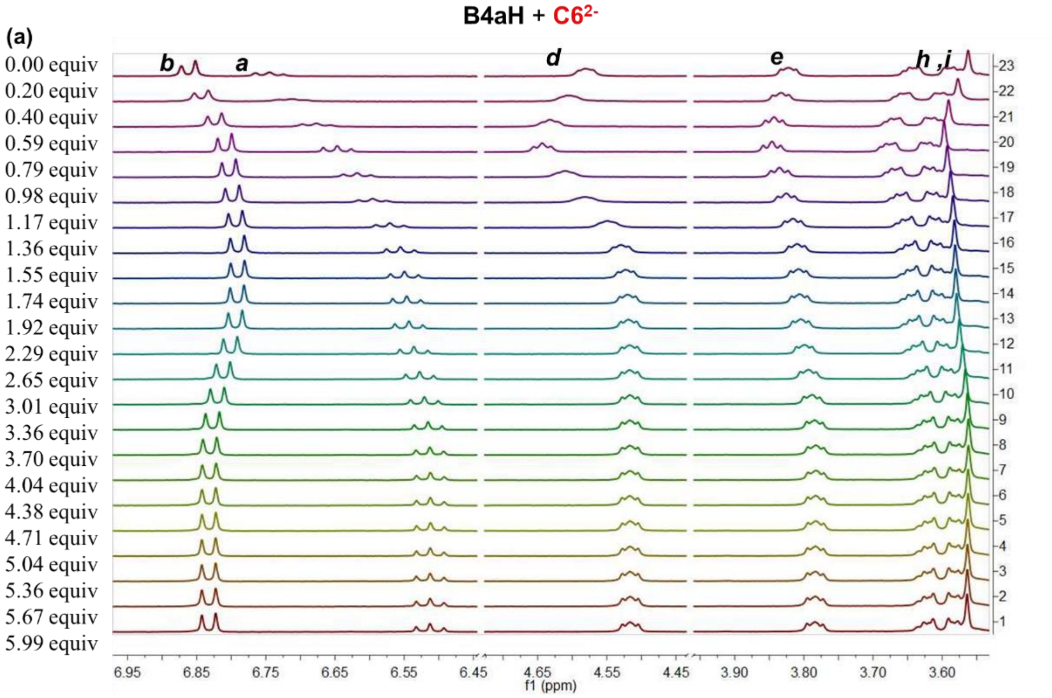

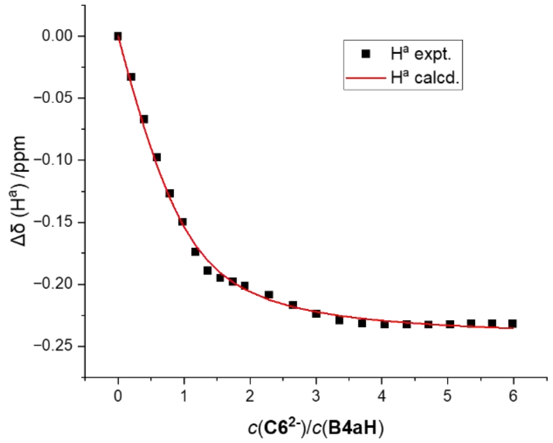

(c)

K_{11} (M^{-1})	4455.8 ± 351.6

Figure S8. (a) ^{1}H NMR titration between **B4aH** and **C6^{2-}** (c(**B4aH**) = 1 mM, c((**TBA$^+$**)$_2$**C6^{2-}**) = 50 mM, 298 K, 400 MHz, CD_3CN); (b) Fitting results of the titration.

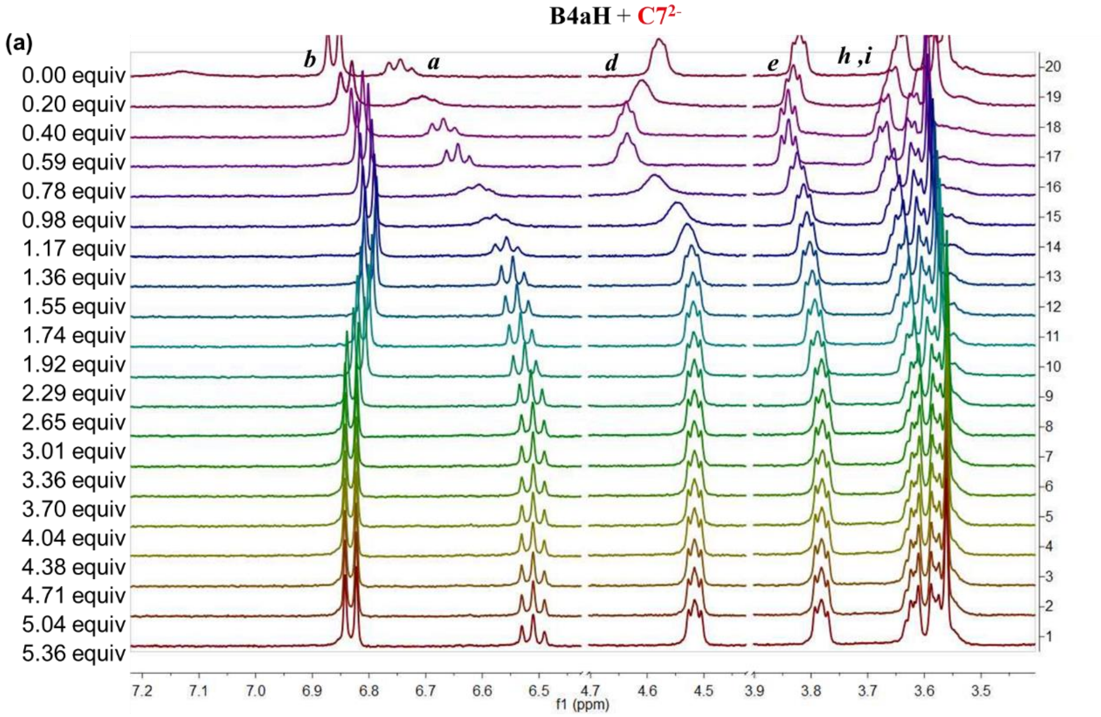

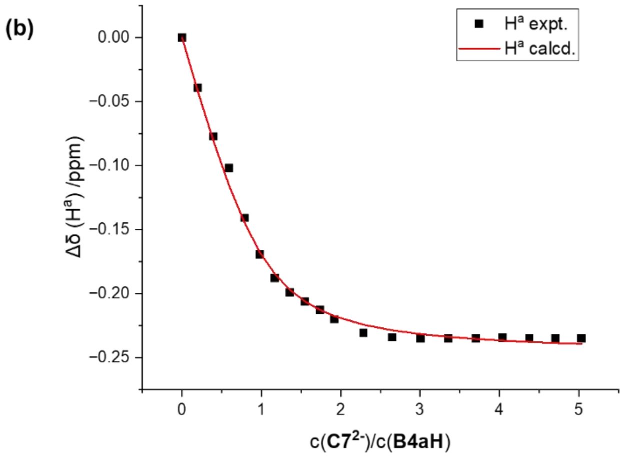

K_{11} (M^{-1})	6896.5 ± 750.1

Figure S9. (a) ^{1}H NMR titration between **B4aH** and **C7**$^{2-}$ (c(**B4aH**) = 1 mM, c((**TBA**$^{+}$)$_2$**C7**$^{2-}$) = 50 mM, 298 K, 400 MHz, CD_3CN); (b) Fitting results of the titration.

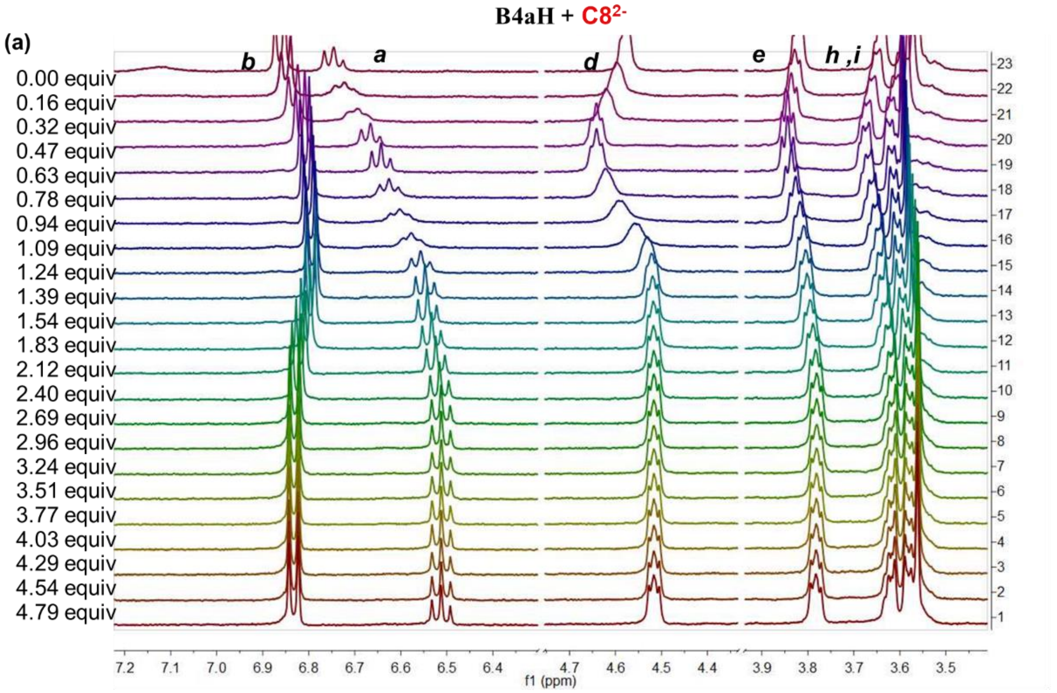

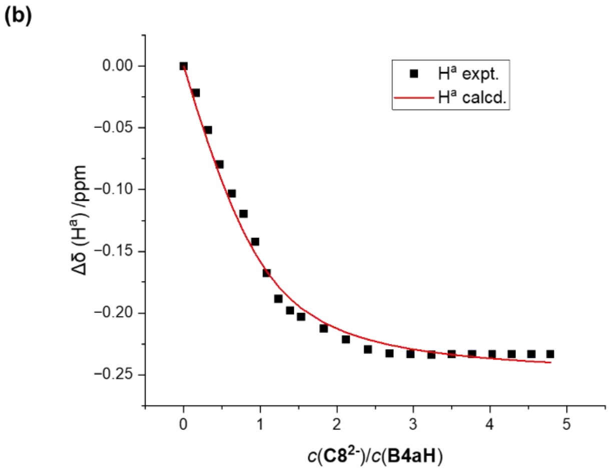

K_{11} (M^{-1})	4423.6 ± 604.3

Figure S10. (a) ^{1}H NMR titration between **B4aH** and **C8**$^{2-}$ (c(**B4aH**) = 1 mM, c((**TBA**$^+$)$_2$**C8**$^{2-}$) = 50 mM, 298 K, 400 MHz, CD_3CN); (b) Fitting results of the titration.

3.2.2 Titration between **B4** and dicarboxylates.

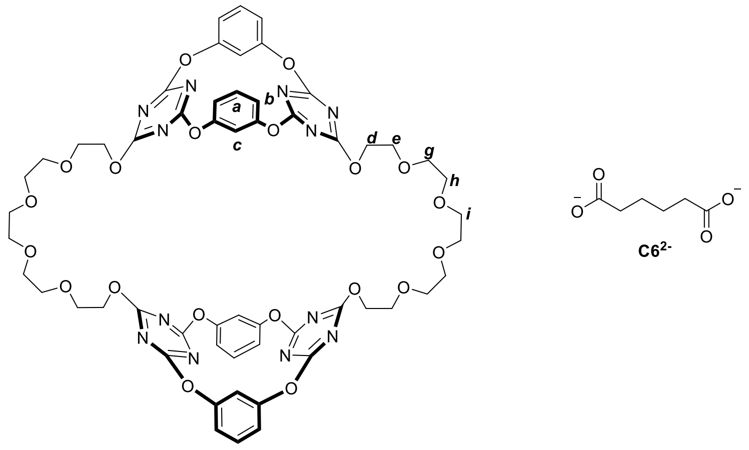

B4

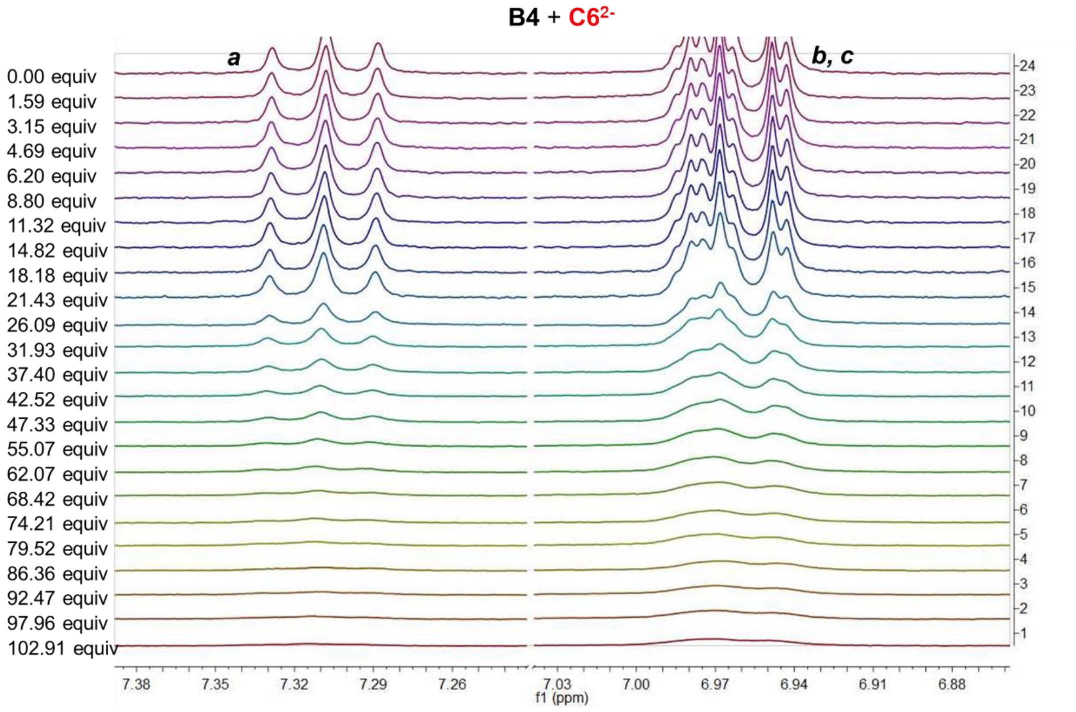

Figure S11. ^{1}H NMR titration between **B4** and **C6**$^{2-}$ (c(**B4**) = 1 mM, c((**TBA**$^{+}$)$_2$**C6**$^{2-}$) = 200 mM, 298 K, 400 MHz, CD_3CN).

4. X-ray diffraction data

Single crystal X-ray diffraction data was collected on a MM007HF Saturn724+ diffractometer using Cu-Kα radiation (λ = 1.54184 Å) at a temperature of 173 K. The intensity data were collected by the omega scans techniques, scaled, and reduced with CrystalClear (Rigaku Inc., 2007). X-ray were provided by a fine-focus sealed X-ray tube operated at 50 kV and 24 mA.

Integrated reflection intensities were produced and correction of collected intensities for absorption was done using the CrystalClear (Rigaku Inc., 2007) program. The structure was solved by direct methods using SHELXT (Sheldrick, 2014/2015). All non-hydrogen atoms were refined anisotropically, and hydrogen atoms attached to carbon atoms were fixed at their ideal positions.

Table S2. Crystal data and structure refinement for **B4aH**.

Identification code	**B4aH**
CCDC number	2427464
Empirical formula	C56 H56 N12 O24
Formula weight	1281.12
Temperature	172.99(10) K
Wavelength	1.54184 Å
Crystal system	Triclinic
Space group	*P*−1
Unit cell dimensions	a = 13.0092(4) Å, α = 65.878(3)°
	b = 15.5860(6) Å, β = 81.112(2)°
	c = 16.2685(4) Å, γ = 69.986(3)°
Volume	2828.36(17) Å^3
Z	2
Density (calculated)	1.504 Mg/m^3
Absorption coefficient	1.021 mm^{-1}
F(000)	1336
Crystal size	0.150 × 0.070 × 0.020 mm^3
Theta range for data collection	2.977 to 76.771°.
Index ranges	-15<=h<=16, -16<=k<=19, -20<=l<=20
Reflections collected	38656
Independent reflections	11378 [R_{int} = 0.0715]
Completeness to theta = 67.684°	99.70%
Absorption correction	Semi-empirical from equivalents
Max. and min. transmission	1.00000 and 0.60597
Refinement method	Full-matrix least-squares on F2
Data / restraints / parameters	11378 / 0 / 833
Goodness-of-fit on F2	1.023
Final R indices [I>2sigma(I)]	R_1 = 0.0526, wR_2 = 0.1436
R indices (all data)	R_1 = 0.0764, wR_2 = 0.1525
Extinction coefficient	n/a
Largest diff. peak and hole	0.708 d −0.438 e·Å^{-3}

5. DFT calculation

Geometrical optimization was carried out with the Gaussian 16 suites[S7] using M06-2X[S8] density functional. 6-31G(d)[S9] basis set was used for all atoms for structure optimization. Frequency analyses were run to confirm that there is no imaginary frequencies and the structure obtained is local minima.

Electronic Energy: −5201.9214

Cartesian coordinates:

C	-3.33066100	-3.88273800	0.34050200	O	-7.03483300	3.45336400	0.64964000
C	-5.93109600	-1.69187600	2.85470700	H	-7.42496700	-2.03562200	4.32953300
O	-6.19763900	-0.33062100	2.85180200	H	-7.55124600	-5.63804200	-2.12441100
N	-7.11672800	-0.52628900	0.75622900	H	-4.84911300	-5.36288000	3.33374900
C	-6.25171700	-3.81644300	3.93872500	H	-9.98725300	-2.19729700	-1.16146200
C	-6.62107700	-2.48899100	3.75937900	H	0.48939100	-3.16429300	-0.63858100
C	-6.68927400	0.25072100	1.74781400	H	-0.56508200	-1.74701500	-0.68342900
N	-6.69820800	1.57037600	1.80383900	H	1.42361700	-0.98025800	-1.73676100
C	-7.12924200	2.12251600	0.67352200	H	0.30754700	-1.34318700	-3.08157000
N	-7.63041800	1.49201700	-0.38140400	H	-6.35256400	3.00622600	-1.96054300
C	-7.56891500	0.16782300	-0.27787100	H	-7.12973600	4.49490000	-2.55846300
C	-2.11609100	-3.58272900	-1.48925000	H	-8.49547500	3.73910300	-0.78549900
N	-3.14235800	-3.85507900	-2.30195100	H	-7.60086500	5.17509500	-0.19501400
C	-4.27351900	-4.04832400	-1.65023900	C	2.49202400	-0.79836900	1.80794100
N	-2.13202400	-3.62820000	-0.15631100	C	6.13337500	-2.92510700	0.30660000
C	-4.87923800	-2.19223200	2.08261200	O	7.14420500	-2.60222400	-0.59010400
C	-7.67672500	-4.58036700	-1.91810900	N	7.80103200	-0.86938100	0.75697700
N	-4.45078200	-4.08676900	-0.33193000	C	5.12261300	-4.54252000	1.75920700
O	-8.06093700	-0.47955800	-1.34083300	C	6.20688300	-4.12264000	0.99033600
O	-5.35735700	-4.27438200	-2.42461000	C	7.72155700	-1.39353800	-0.46238600
O	-3.37456000	-3.99285600	1.68735200	N	8.17655400	-0.87782900	-1.59281300
C	-4.51675800	-3.53026800	2.30883400	C	8.68657100	0.34217600	-1.42850900
C	-5.18359900	-4.33855900	3.21135400	N	8.89900900	0.96178000	-0.26934700
C	-8.92400000	-4.03129400	-1.61797700	C	8.40914500	0.30422000	0.77399300
C	-9.03352500	-2.66512200	-1.38326800	C	1.04317200	0.69188100	2.59790400
C	-6.56457600	-3.76177200	-1.97794000	N	1.99389100	1.38404200	3.22923300
C	-7.90423200	-1.86044800	-1.43985000	C	3.20079600	0.87628400	3.06300800
C	-6.63518000	-2.37625300	-1.73074500	N	1.22415300	-0.40057400	1.85333500
C	-0.04003000	-2.47855600	-1.30675000	C	5.00733200	-2.07639100	0.35542900
C	0.89883700	-1.78400300	-2.27210400	C	5.84979100	0.59066700	5.00508200
C	-6.58614000	4.04984700	-1.70570800	N	3.53026800	-0.23491000	2.40528500
C	-7.51312000	4.13307300	-0.51127200	O	8.60985100	0.91422800	1.95328800

O	4.17456700	1.58092700	3.65525500	C	7.85578100	3.14863700	-1.96137800
O	2.69091100	-1.90413000	1.07469000	O	6.71233400	2.84304000	-2.71041700
C	3.92576400	-2.54758600	1.11573300	C	5.83325300	1.89242400	-2.11332300
C	3.98226100	-3.75134000	1.80845200	C	5.27702900	0.99211400	-3.19046600
C	7.15929900	0.18698500	5.22945800	O	6.38250600	0.39615100	-3.84022800
C	8.07551100	0.25704900	4.18197000	C	6.06954600	-0.63969800	-4.72880600
C	5.45222400	1.04618400	3.75340700	C	5.79481100	-1.96913800	-4.04810300
C	7.66119600	0.71001800	2.94498900	O	4.48396000	-1.96797400	-3.53826800
C	6.33626200	1.10343700	2.66700300	C	4.17254600	-3.15129000	-2.84781900
O	-5.40867700	4.78318100	-1.44687800	C	2.93128700	-2.91335400	-2.02310200
C	-4.69305600	5.00876100	-2.63997200	O	1.83175100	-2.65939500	-2.86791100
C	-3.28019600	5.44783800	-2.33308200	H	9.28309400	2.70256100	-3.51272100
O	-3.28411600	6.75552700	-1.78713000	H	9.96277200	2.68042300	-1.85612800
C	-2.45247500	6.91773900	-0.66216800	H	8.06498700	4.22217600	-2.07618200
C	-3.10189300	6.44498000	0.62410500	H	7.70227100	2.95276200	-0.89513900
O	-3.09699300	5.04430600	0.69286200	H	5.02134600	2.41055400	-1.58672500
C	-3.74957500	4.59754200	1.86101800	H	6.35401200	1.27605500	-1.36967300
C	-3.81353700	3.09365800	1.91363900	H	4.69199100	1.57964200	-3.91790700
O	-2.51109000	2.54979900	1.96995100	H	4.61747800	0.24895900	-2.72883700
C	-2.54305000	1.22048400	2.44479900	H	6.94790900	-0.75580800	-5.37475600
C	-1.26145100	0.50699400	2.10025700	H	5.20566100	-0.37727300	-5.36226100
O	-0.17917300	1.16975400	2.76894400	H	5.91398000	-2.78824600	-4.78215100
H	7.10907300	-4.71737000	0.89369200	H	6.52859300	-2.11057000	-3.24032600
H	5.10600000	0.58200900	5.79523800	H	4.99485200	-3.42965000	-2.16912500
H	3.10032700	-4.05455900	2.36328200	H	4.01726200	-3.98021500	-3.55932400
H	9.11579200	-0.02522300	4.30504700	H	3.11620800	-2.04159100	-1.38654000
H	-5.20000300	5.78348700	-3.24085100	H	2.74982000	-3.78411300	-1.37137000
H	-4.65612400	4.08921000	-3.24224900	O	-1.00446600	-3.20279800	-2.09668600
H	-2.70073300	5.44678400	-3.26951800	H	-6.78648800	-4.44020000	4.64715700
H	-2.82602800	4.73684400	-1.63903700	H	-9.80268700	-4.66569000	-1.57040300
H	-2.25469500	7.99268400	-0.57971500	H	5.16302700	-5.48108100	2.30102900
H	-1.49078700	6.40144900	-0.79686700	H	7.46555400	-0.16965500	6.20692800
H	-4.13536300	6.82801200	0.65302600	O	5.05020900	-0.96582000	-0.36320500
H	-2.55491000	6.86914200	1.48547200	H	4.21919400	-0.33981000	-0.32853200
H	-4.78251000	4.98113500	1.88091500	O	6.06816200	1.52464300	1.43959000
H	-3.21598500	4.98072100	2.74676600	H	5.08486300	1.71076700	1.20584100
H	-4.38808100	2.81811500	2.81228800	O	-5.51783600	-1.67552000	-1.79169900
H	-4.36085400	2.69401700	1.05298500	H	-5.59846100	-0.64806500	-1.94642300
H	-3.36236800	0.66369400	1.97722700	O	-4.12082500	-1.49216500	1.23319100
H	-2.69332200	1.21678900	3.53708800	H	-4.56168500	-0.77551500	0.69146900
H	-1.08162700	0.53401400	1.02557700	C	-1.97425100	1.34927600	-1.43624000
H	-1.31304500	-0.54061700	2.41356300	C	-0.83633500	2.29633100	-1.05403300
O	8.96264100	0.99367900	-2.55079900	C	0.51697800	1.58619300	-0.98185600
C	9.09823400	2.41621900	-2.47557000	C	1.59240200	2.40416500	-0.27746100

C	-3.32977900	2.06002900	-1.57189700	H	0.85885600	1.31436900	-1.98831000
C	-4.47225800	1.09522600	-1.28436900	H	1.82890300	3.31778600	-0.83949700
O	-4.56040300	0.68097800	-0.09764300	H	1.23960500	2.74851100	0.70345200
O	-5.23837200	0.75832100	-2.23031300	H	-3.44140700	2.47979700	-2.57552600
H	-2.07706700	0.58097600	-0.66091900	H	-3.38362800	2.87192500	-0.83610400
H	-1.73606700	0.81805600	-2.36785000	C	2.90918300	1.66430300	-0.04053100
H	-0.78200900	3.12600400	-1.77603600	O	3.74730100	2.24663900	0.69267800
H	-1.08353200	2.74593200	-0.08174800	O	3.05363100	0.53097200	-0.58496200
H	0.40847700	0.63526000	-0.44301800				

6. Copies and ^{1}H and ^{13}C NMR spectra

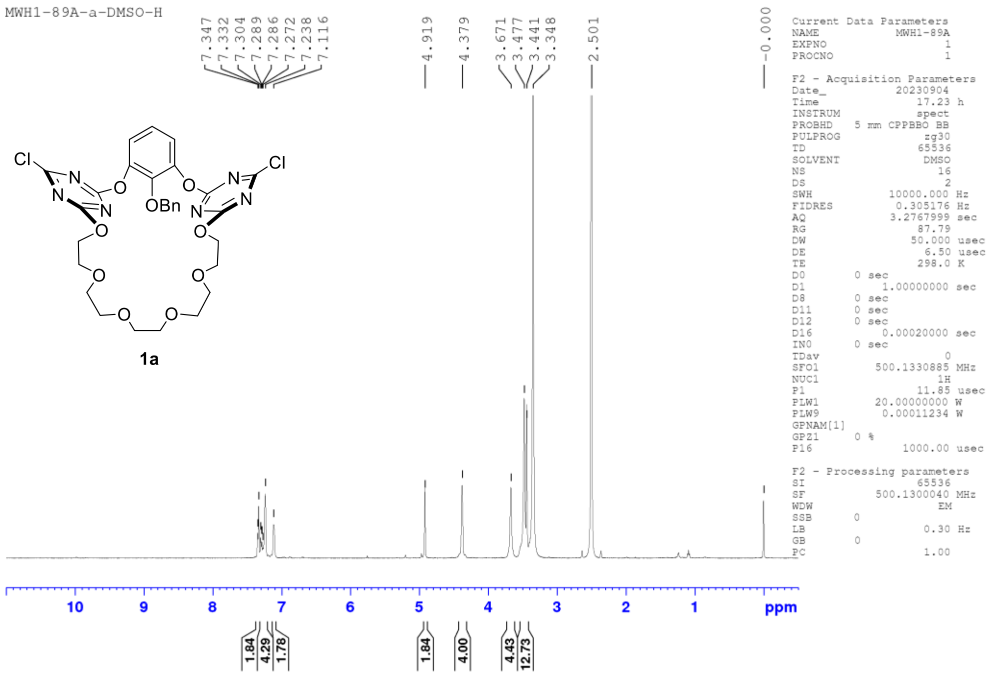

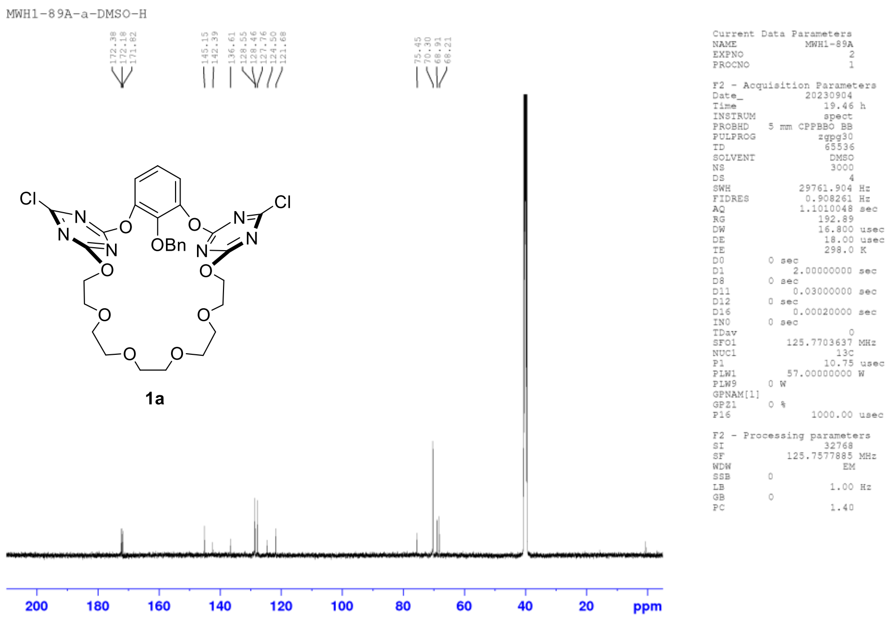

^{1}H and ^{13}C NMR of **1a** in DMSO-d_6

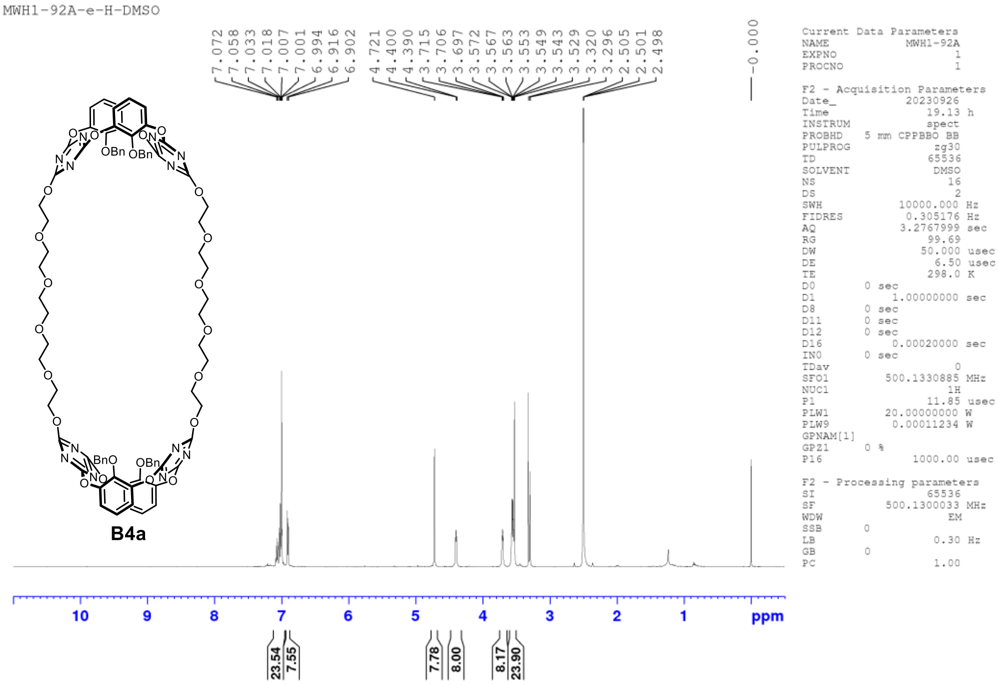

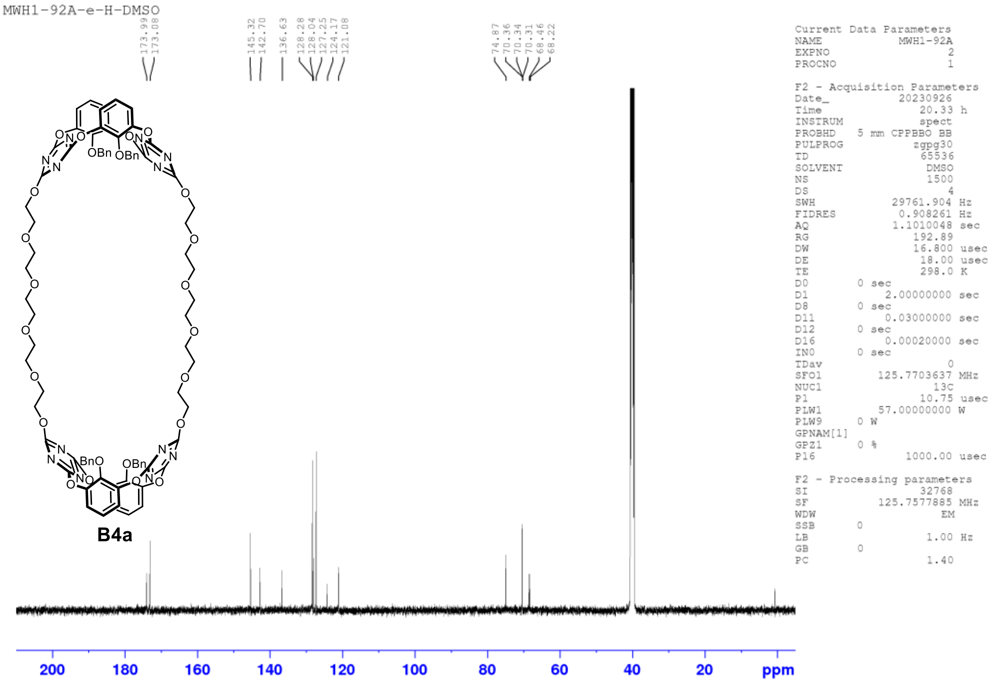

^{1}H and ^{13}C NMR of **B4a** in DMSO-d_6

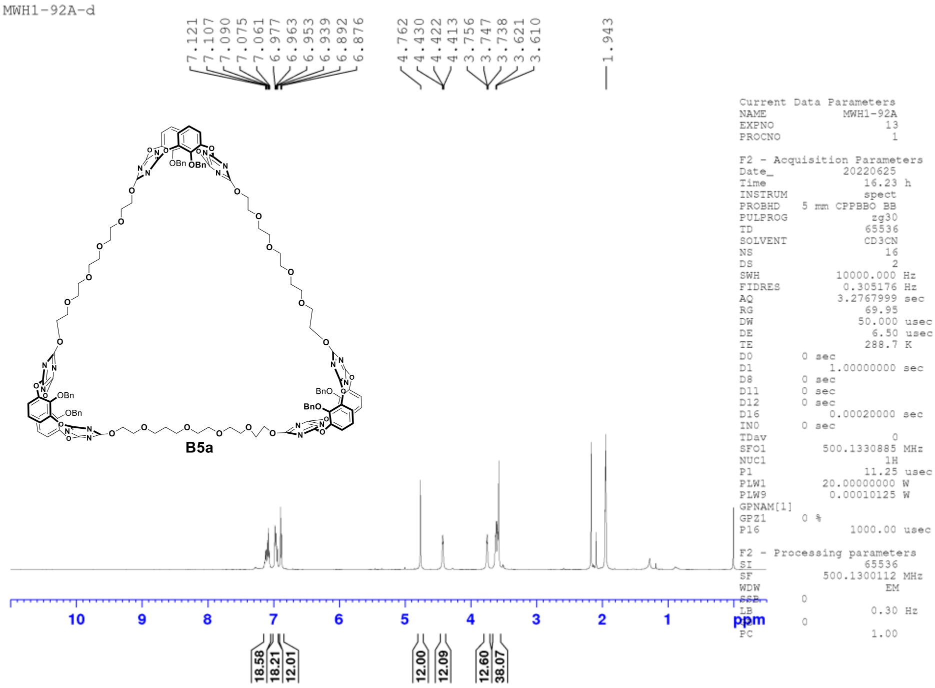

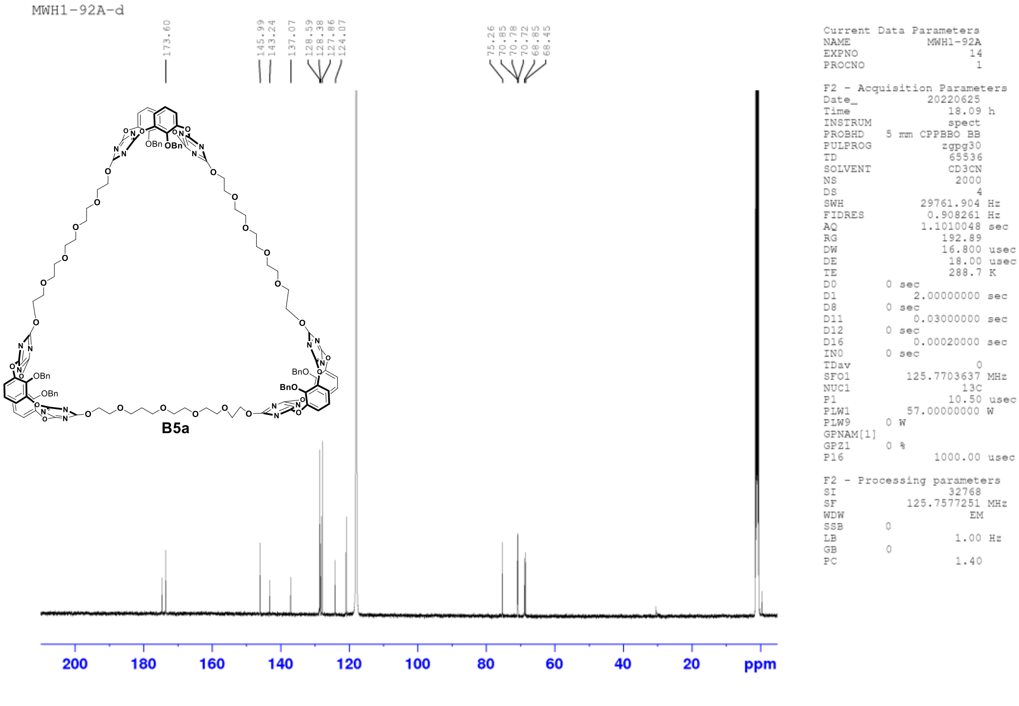

^{1}H and ^{13}C NMR of **B5a** in CD_3CN

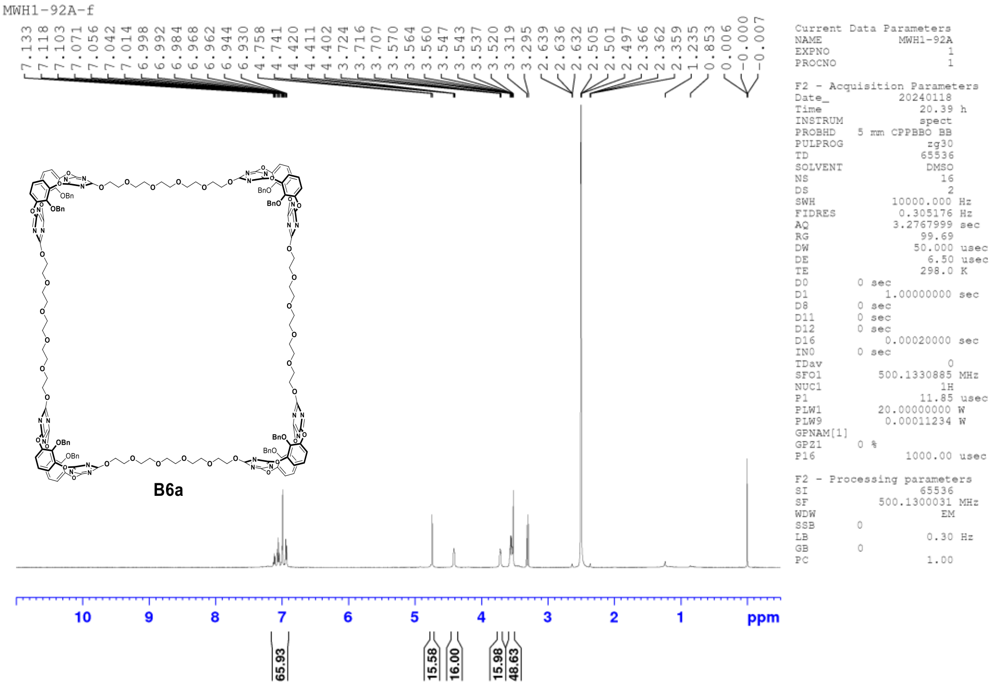

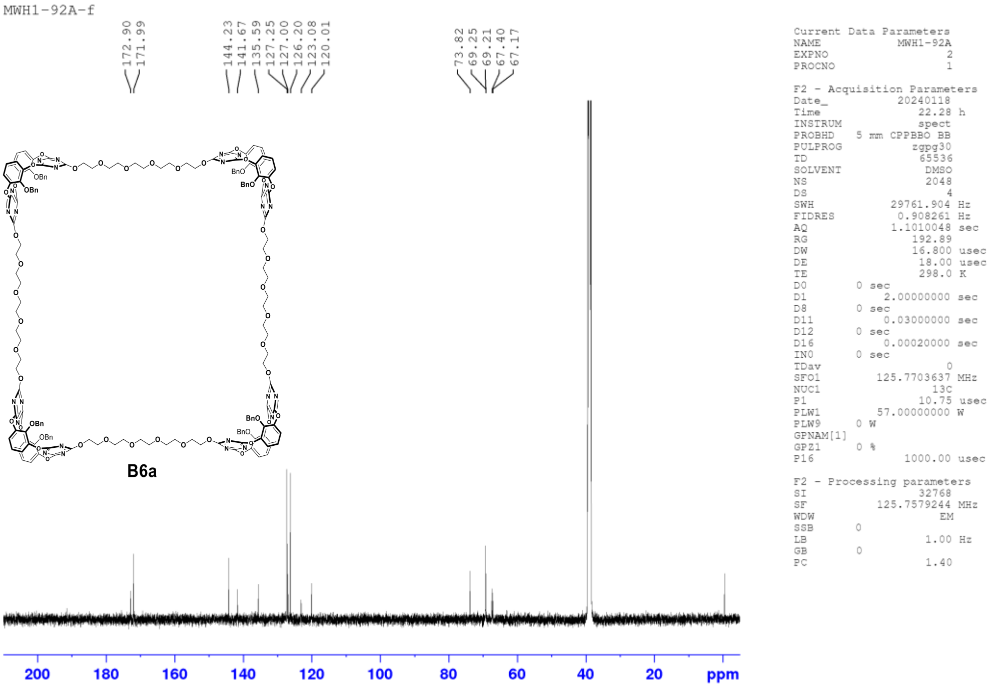

^{1}H and ^{13}C NMR of **B6a** in DMSO-d_6

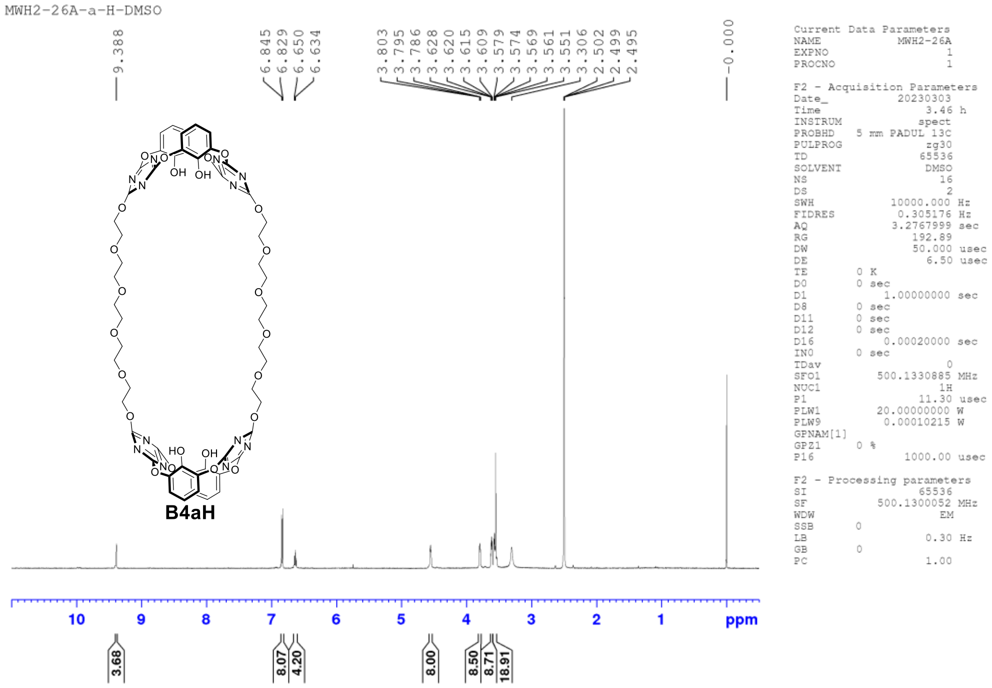

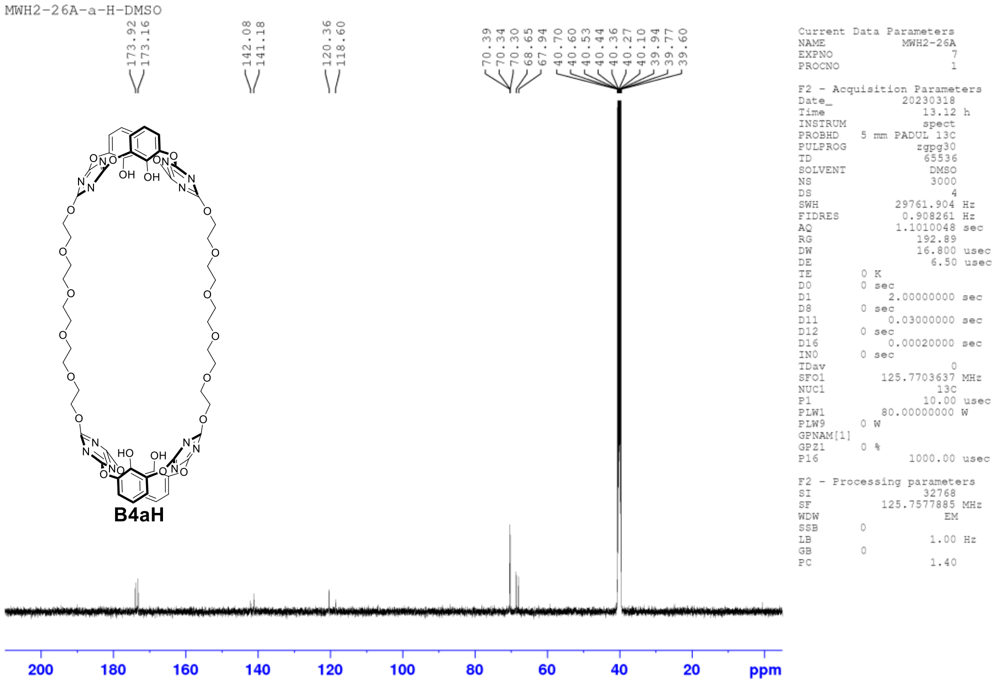

^{1}H and ^{13}C NMR of **B4aH** in DMSO-*d*₆

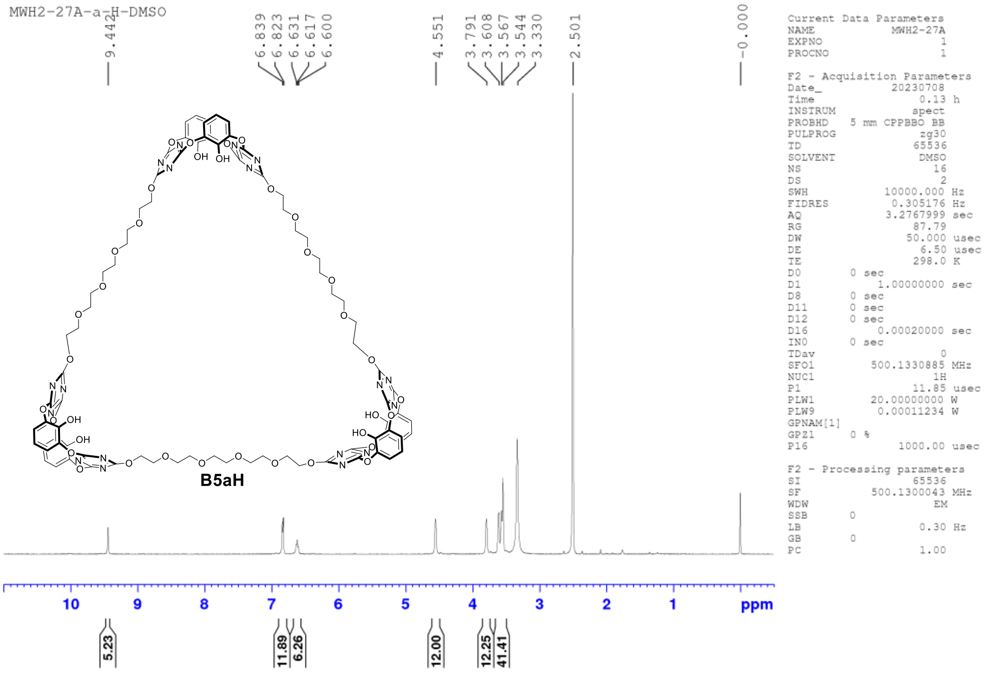

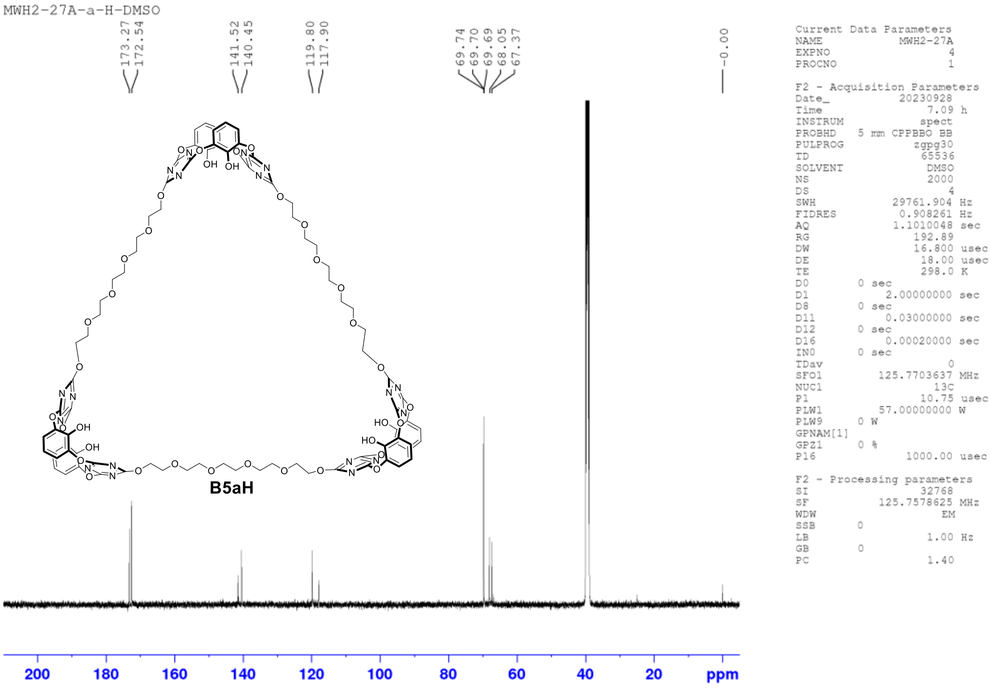

^{1}H and ^{13}C NMR of **B5aH** in DMSO-d_6

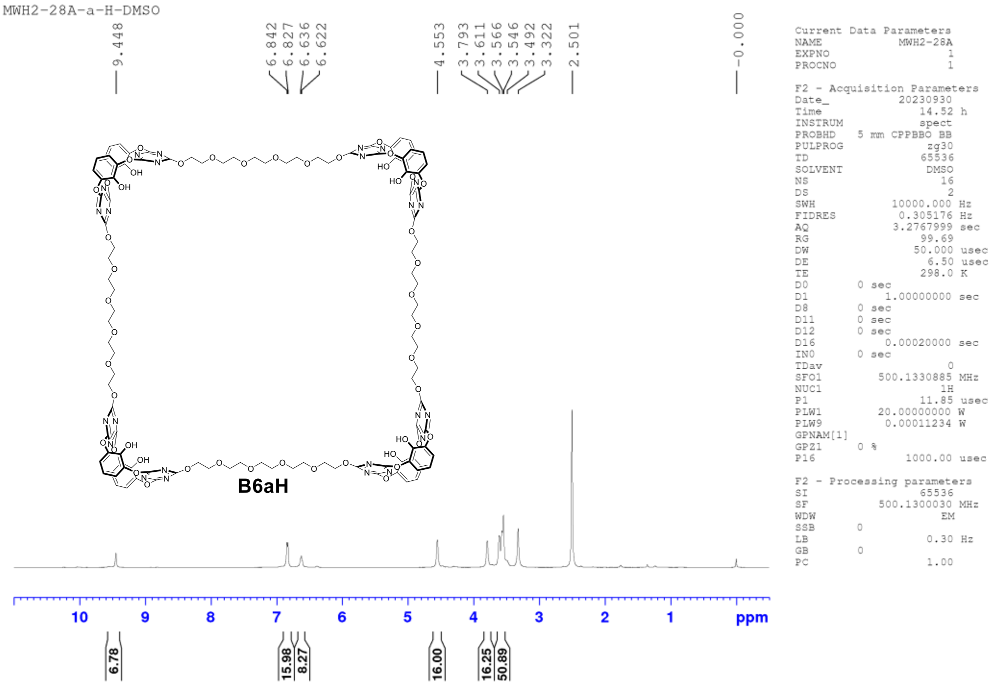

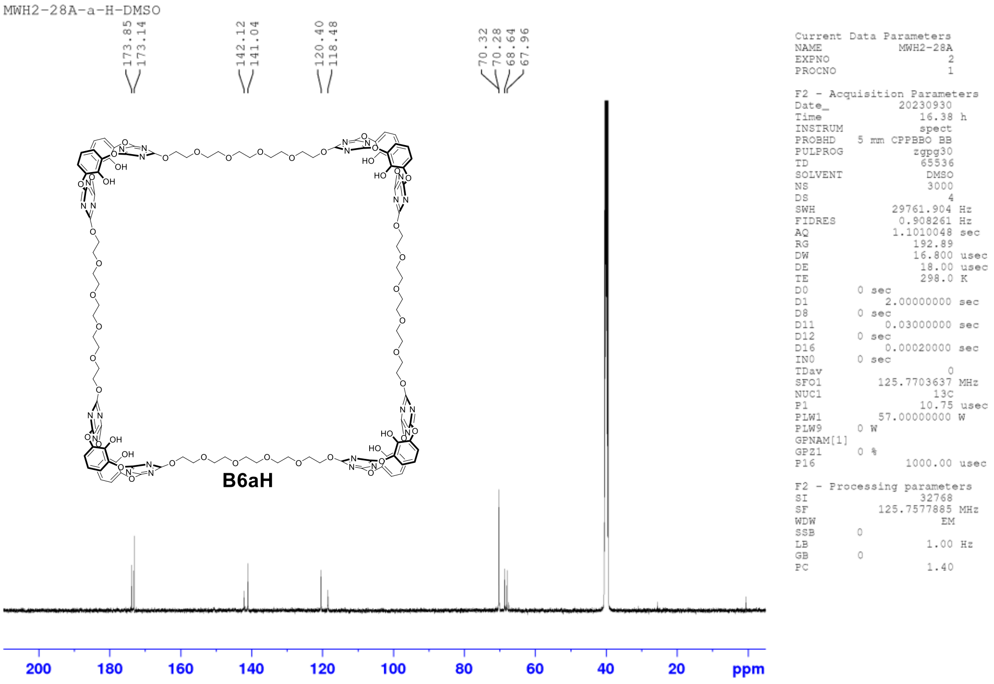

^{1}H and ^{13}C NMR of **B6aH** in DMSO-d_6

7. References

[S1] Luo, J.; Ao, Y.-F.; Malm, C.; Hunger, C.; Wang, Q.-Q.; Wang, D.-X. *Dalton Trans*. **2018**, *47*, 7883-7887. doi: 10.1039/C8DT01727A.

[S2] Luo, J.; Ao, Y.-F.; Wang, Q.-Q.; Wang, D.-X. *Angew. Chem. Int. Ed.* **2018**, *57*, 15827–15831. DOI: 10.1002/ange.201810836.

[S3] Michuhata, N.; Kaneko, Y.; Kasai, Y.; Tanigawa, K.; Hirokane, T.; Higasa, S.; Yamada, H. *J. Org. Chem.* **2013**, *78*, 4319–4328. DOI: 10.1021/jo4003135.

[S4] *Bindfit v0.5*: Supramolecular.org. http://supramolecular.org.

[S5] Thordarson, P. *Chem. Soc. Rev*. **2011**, *40*, 1305-1323. DOI: 10.1039/c0cs00062k.

[S6] Hibberta, D. B.; Thordarson, P. *Chem. Commun.* **2016**, *52*, 12792-1280. DOI: 10.1039/c6cc03888c.

[S7] Frisch, M.J.; Trucks, G.W.; Schlegel, H.B.; Scuseria, G.E.; Robb, M.A.; Cheeseman, J.R.; Scalmani, G.; Barone, V.; Mennucci, B.; Petersson, G.A.; Nakatsuji, H.; Caricato, M.; Li, X.; Hratchian, H.P.; Izmaylov, A.F.; Bloino, J.; Zheng, G.; Sonnenberg, J.L.; Hada, M.; Ehara, M.; Toyota, K.; Fukuda, R.; Hasegawa, J.; Ishida, M.; Nakajima, T.; et al.; Gaussian 16, Revision A. 03; Gaussian, Inc.: Wallingford, CT, 2016.

[S8] Petersson, G. A.; Bennett, A.; Tensfeldt, T. G.; AI-Laham, M. A.; Shirley, W. A.; Mantzaris, J. *J. Chem. Phys.*, **1988**, *89*, 2193–2218. DOI: 10.1063/1.455064 .

[S9] Zhao, Y.; Truhlar, D. G. *Theor. Chem. Acc.*, **2008**, *120*, 215–241. DOI: 10.1007/s00214-007-0310-x.
